# Psychiatric medications and the risk of autoimmune and immune-mediated inflammatory diseases: A systematic review and meta-analysis of observational studies

**DOI:** 10.1371/journal.pone.0281979

**Published:** 2023-02-28

**Authors:** Ilmari Larivuo, Heidi Laukkala, Anna Nevalainen, Otso Arponen, Olli P. O. Nevalainen

**Affiliations:** 1 Faculty of Social Sciences, Tampere University, Tampere, Finland; 2 Hatanpää Health Center, Wellbeing Services County of Pirkanmaa, Tampere, Finland; 3 Groningen Research Institute of Pharmacy, University of Groningen, Groningen, Netherlands; 4 Faculty of Medicine and Health Technology, Tampere University, Tampere, Finland; 5 Department of Radiology, Tampere University Hospital, Tampere, Finland; Chiba Daigaku, JAPAN

## Abstract

**Background:**

Pharmacovigilance reports have suggested that certain commonly used medications may trigger autoimmune diseases (ADs) and immune-mediated inflammatory diseases (IMIDs). We systematically reviewed the literature to evaluate whether psychiatric medication use is associated with ADs and IMIDs.

**Methods:**

The protocol was registered in PROSPERO (CRD42022296524) before the start of the study. We searched Medline Ovid and Scopus up to November 28^th^, 2021, for comparative studies, with any psychiatric medication as exposure and ADs and IMIDs as outcomes. Meta-analysis was performed using DerSimonian-Laird random-effects modeling. The PRISMA 2020 guidelines were followed in reporting. Study-level risk of bias was assessed using the Newcastle-Ottawa Scale, and the overall certainty of evidence using GRADE.

**Results:**

There were 7,265 citations from which 31 studies were eligible, all from high-income countries, covering 15 distinct immune diseases. The evidence for the association between selective serotonin reuptake inhibitor (SSRI) use and higher risk of microscopic colitis (meta-OR 2.60, 95% CI 1.05–6.39, I^2^ 97.5%, 6 studies) was of low certainty. A subgroup analysis by the histological type of microscopic colitis showed a statistically significant association only with lymphocytic colitis (meta-OR 2.88, 95% CI 2.60–3.18, I^2^ 00.00%, three studies). In two case-control studies, SSRI use had no significant association with psoriasis (meta-OR 0.80, 95% CI 0.58–1.10, I^2^ 82.4%). The risk of acute pancreatitis was slightly increased with exposure to SSRIs (meta-OR 1.13, 95% CI 1.01–1.26, I^2^ 00.0%), as was the risk of bullous pemphigoid after exposure to antipsychotics (meta-OR 1.79, 95% CI 1.17–2.73, I^2^ 0%).

**Conclusions:**

We reviewed the literature on whether psychiatric medications associate with the risk of ADs and IMIDs and concluded that, despite several signals, the credibility of evidence remains low at best. Prospective cohort studies would be needed as the next step to confirm the suggestions of increased risk.

## Introduction

The causes of most autoimmune diseases (ADs) and immune-mediated inflammatory disorders (IMIDs) are unknown. The etiology and pathophysiology of most of them are probably unrelated, yet some share common genetic and environmental risk factors. The common denominator of these diseases is the dysregulation of immune pathways, leading to mistargeted or excessively activated responses, manifesting in subsequent organ-specific or multisystem damage [1]. Globally, the incidence of many of these diseases is increasing, including celiac disease, rheumatoid arthritis, inflammatory bowel diseases, and type 1 diabetes mellitus [2]. Numerous case reports, case series, and some pharmacovigilance risk signals suggest that certain commonly prescribed medications could trigger immune diseases [3]. Additionally, some observational studies suggest that exposure to antidepressants may be associated with a beneficial course in certain inflammatory diseases [4].

The most widely documented drug-induced autoimmune phenomenon is drug-induced lupus (DIL). A range of medications has been linked with it, including psychiatric drugs such as carbamazepine and levomepromazine [5]. Drug-induced immune thrombocytopenia characteristically occurs within two weeks after initiation of the causative medication and re-occurs rapidly after subsequent exposures [6]. Among inflammatory diseases without autoimmune mechanisms, the United European Gastroenterology organization and the European Microscopic Colitis Group have both stated that the use of selective serotonin reuptake inhibitors (SSRIs) is associated with an increased risk of developing an inflammatory colonic disease called microscopic colitis (MC) [3].

Animal models have associated mental stress with a proinflammatory state [7]. In humans, there is a relationship between having mental disorders and higher levels of proinflammatory cytokines (e.g., TNF-alpha, interleukin-6) that can activate the inflammatory response system [7]. This relationship appears bidirectional, as higher levels of these cytokines also increase the risk of depression [8].

We aimed to assess whether reports from case series could be supported by epidemiological evidence from comparative observational studies. Due to uncertainty in the etiology of the range of these diseases and finding no biological rationale to restrict the search to only a few of these diseases, we conducted a search of all major ADs and IMIDs covered in the literature.

## Methods

The systematic review protocol was registered on PROSPERO (CRD42022296524) before commencing the study. The Preferred Reporting Items for Systematic Reviews and Meta-Analyses (PRISMA) 2020 statement was used to guide the reporting of the process [9]. Authors IL and ON searched MEDLINE Ovid and Scopus from inception until 28 November 2021 (non-duplicate process). After this initial search of titles and abstracts, full-texts were read independently and in parallel by IL and ON. Reference lists of eligible articles were browsed for potentially missed studies. Studies were eligible if they satisfied the following inclusion criteria, which are demonstrated under the PICO model for cohort studies in Table 1. The full search strategy and PRISMA checklist are available in the supplement. In short, we listed as exposures all psychiatric medications that have been widely used. We included studies having any immune diseases as outcomes after exposure to psychiatric medications. Primarily allergic immune diseases, immune deficiencies, or diseases with an infectious etiology such as viral hepatitis, were not included as outcomes.

**Table 1:** Search question in terms of PICO (Population, intervention, comparison, and outcome).

Population (P)	Human subjects who were exposed to any medication used to treat psychiatric disorders as indexed in the WHO ATC (S4 File). The exception was benzodiazepines, which were not considered. We accepted the drug exposure period as defined by the investigators. Exposure to the medication should have occurred prior to the immune disease diagnosis, and studies with an unclear sequence of events were not considered.
Intervention (I)	We included only observational studies. Because immune diseases are rare, we did not expect to find interventional studies on these relationships.
Comparison (C)	The observational study should include a comparison group of individuals who have not been exposed to or have been exposed to a lesser degree (e.g., regular vs. non-regular use) to the medication. Case series were not included due to the lack of a comparison group.
Outcome (O)	The outcome is the diagnosis of any AD or IMID as has been listed in the search strategy (Supplement). Inflammatory conditions with a clear infectious etiology, such as viral hepatitis, were not included.

We considered original articles published in any language but excluded conference abstracts and reviews. We managed the citations using the RefWorks legacy edition. All authors participated in the data collection and risk-of-bias assessments, which were performed in duplicate by two authors using a Microsoft Excel sheet. The collected data were checked and synthesized by one author (ON). Data items included basic study details, which diseases were studied as outcomes, which medications were studied as exposures, drug exposure definitions, study country and continent, age and gender distributions, years of recruitment, study design (case-control or cohort), and whether the study was prospective or retrospective. Studies were excluded, if an effect measure was not reported and it was not possible to calculate it based on the reported data. Individual studies were assessed with the Newcastle–Ottawa Scale (NOS) in three domains: selection, comparability, and outcome ascertainment [10]. The NOS score ranges from 0 to 9, where 9 indicates the lowest risk of bias. NOS scores are shown in the supplement, including which studies had a sufficient adjustment for confounders. To evaluate the overall strength of evidence of each meta-analysis, we applied the Grading of Recommendations, Assessment, Development, and Evaluations (GRADE) procedure [11].

### Statistical analysis

The summary measure for binary outcomes is the odds ratio (OR) in case-control studies, the risk ratio (RR) in cohort studies, and the hazard ratio (HR) in cohort studies with time-to-event data. RStudio (version 2022.02.2) and the *meta* package with *metagen* and *forest*.*meta* functions were used to perform random-effects meta-analyses (DerSimonian and Laird) and create forest plots. Between-study heterogeneity in estimates is designated by the Tau^2^ and I^*2*^ statistics, and in the case of the latter, 0–40% indicates non-important heterogeneity, and 75–100% indicates very important heterogeneity [12, 13]. We used the harvest plot to visualize the results when it was not possible to conduct a meta-analysis [14]. Reanalyzing the data using different statistical approach was used for the sensitivity analysis in the current study.

## Results

**Fig 1:**
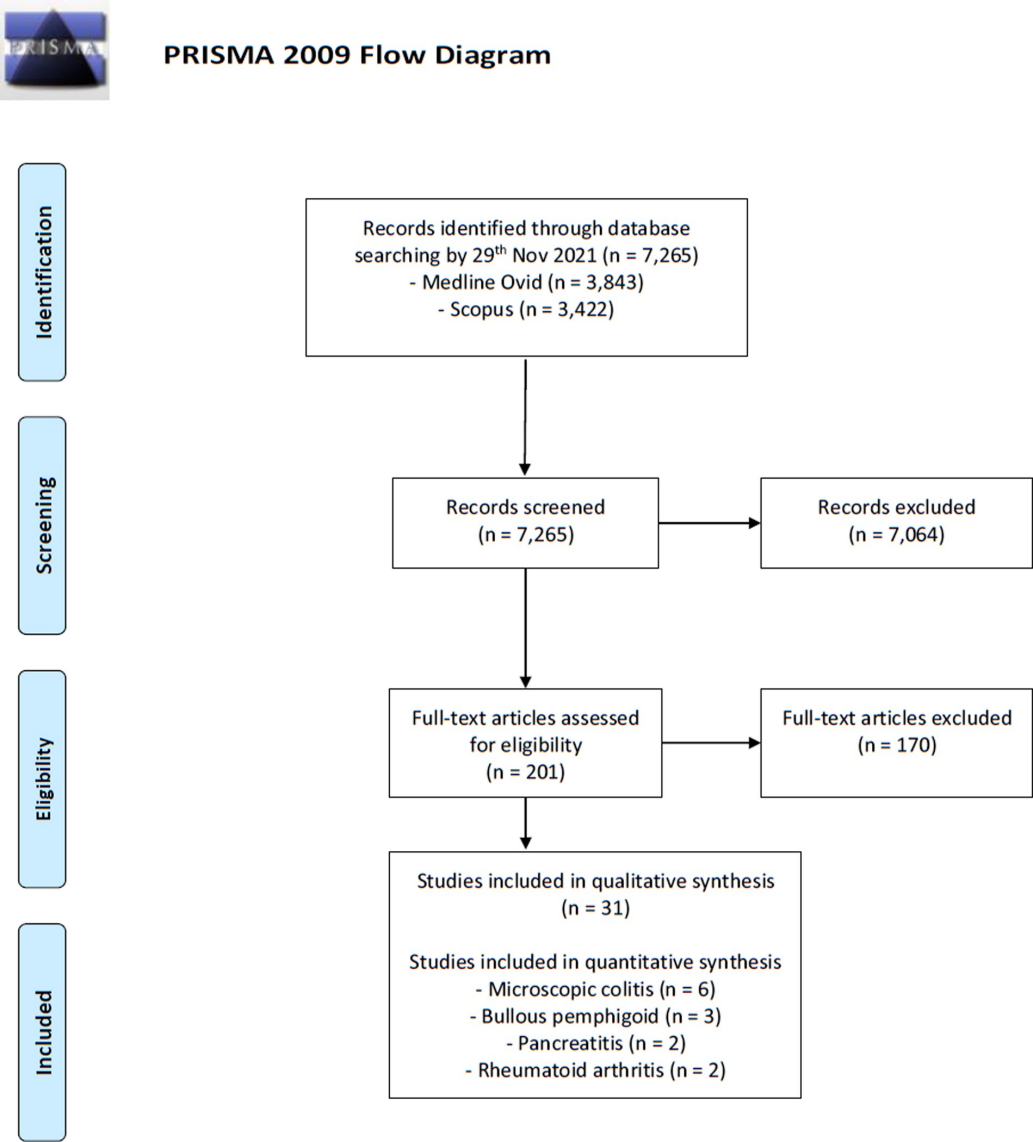
Flowchart of study selection.

The primary search yielded 7,265 citations, of which 203 were assessed in duplicate (authors IL and ON), eventually contributing 31 articles eligible for narrative review (Fig 1, Table 2) [15–45]; seven on MC [15–21], six on acute pancreatitis (AP) [22–27], four on bullous pemphigoid [28–31], three on lupus erythematosus [32–34], two on alopecia areata [35, 36], psoriasis [37, 38], and rheumatoid arthritis [39, 40], while one on immune thrombocytopenia [41], cutaneous lupus erythematosus [34], interstitial lung disease [42], multiple sclerosis [43], silent thyreoiditis [44], and vitiligo [45]. According to the World Bank income classification, all studies were conducted in countries of high income. Studies from Europe comprised the majority, with 74% (23/31), followed by 19% (6/31) from Northern America, and the two Asian studies were from Taiwan (Table 1).

**Table 2:** Characteristics of studies reporting an association between the exposure to psychiatric medications and the risk of autoimmune diseases or immune-mediated inflammatory diseases.

Author, year	Country, continent	Disease(s)	Individual medications or groups analyzed	Age distribution, years	Years of recruitment	Cohort or case-control	Prospective or retrospective
Bonderup 2014	Denmark, Europe	MC, CC, and LC	(1) SSRI	Mean 64.7 (CC), 63.9 (LC)	2005–2011	Case-control	Prospective
Fernández-Bañares 2013	Spain, Europe	MC, CC, and LC	(1) Sertraline	Mean 62.5 ± 1.4 (CC), 62.6 ± 1.9 (LC)	3/2007–5/2010	Case-control	Prospective
Fernández-Bañares 2007	Spain, Europe	MC, CC, and LC	(1) SSRI	Mean 61.0 ± 2.02 (CC), 67.4 ± 2.25 (LC)	1/1993–12/1999	Case-control	Prospective
Masclee 2015	Netherlands, Europe	MC	(1) SSRI	Mean 45.1 ± 2.1	1/1999–3/2012	Nested case-control	Prospective
Pascua 2010	US, North America	MC	(1) SSRI	Median 68.9, range 18–92	2002–2007	Case-control	Retrospective
Verhaegh 2016	UK, Europe	MC, CC, and LC	(1) SSRI	Mean 63.4 ± 14	1/1992–12/2013	Case-control	Retrospective
Weimers 2021	Denmark, Europe	MC	(1) SSRI	Mean 65.0 ± 14.0	1/1995–12/2016	Case-control	Retrospective
Boden 2012	Sweden, Europe	AP	(1) Clozapine & olanzapine (2) Other antipsychotics: fluphenazine, perphenazine, flupenthixol, thioridazine, chlorpromazine, haloperidol, pimozide, ziprasidone, aripiprazole, quetiapine, risperidone, paliperidone, clozapine, and olanzapine	Range 40–85	1/2006–12/2008	Nested case-control	Retrospective
Gasse 2008	Denmark, Europe	AP	(1) Conventional, including flupentixol, fluphenazine, haloperidol, pimozide, penfluridol, periciazine, perphenazine, prochlorperazine, zuclopenthixol, chlorpromazine, chlorprothixene, levomepromazine, melperone, and thioridazine (2) Atypical antipsychotics: clozapine, olanzapine, risperidone, quetiapine, ziprasidone, sulpiride, levosulpiride, and amisulpride	Range 18–89	1991–2003	Case-control	Retrospective
Lin 2017	Taiwan, Asia	AP	(1) SSRI (2) Non-SSRI antidepressants	Mean 50.9 ± 15.9, range 20–84	2000–2013	Case-control	Retrospective
Ljung 2012	Sweden, Europe	AP	(1) SSRI (2) Non-SSRI	Range 40–84	2006–2008	Case-control	Retrospective
Norgaard 2006	Denmark, Europe	AP	(1) Valproic acid	Range 18–89	North Jutland 1991–2003, Aarhus 1996–2003, and Viborg 1998–2003	Case-control	Retrospective
Norgaard 2007	Denmark, Europe	AP	(2) SSRI	Range 18–89	North Jutland 1991–2003, Aarhus 1996–2003, and Viborg 1998–2003	Case-control	Retrospective
Bastuji-Garin 1996	France, Europe	BP	(1) Neuroleptics	Mean 79.2 ± 10.1	3/1989–2/1992	Case-control	Prospective
Bastuji-Garin 2011	France, Europe	BP	(1) Neuroleptics (N05A) (2) Psycholeptic agents (N05) (3) Phenothiazine aliphatic chain (N05AA) (4) Psychoanaleptics (N06)	Mean 82.4 ± 8.7	1/2003–4/2007	Case-control	Prospective
Lloyd-Lavery 2013	UK, Europe	BP	(1) Antipsychotics (2) Antidepressants (3) SSRI (4) TCA (5) SNRI (6) Mirtazapine (7) Tradozone	Mean 81.5 ± 9.7	1/2004–12/2008	Case-control	Retrospective
Varpuluoma 2019	Finland, Europe	BP	(1) Carbamazepine (2) Pregabalin (3) Biperiden (4) Levomepromazine (5) Perphenazine (6) Periciazine (7) Haloperidol (8) Melperone (9) Quetiapine (10) Sulpiride (11) Risperidone (12) Hydroxyzine (13) Amitriptyline (14) Doxepin (15) Citalopram (16) Sertraline (17) Escitalopram (18) Mianserin (19) Mirtazapine (20) Venlafaxine (21) Duloxetine	Mean 76.6	1987–2013	Case-control	Retrospective
Grönhagen 2012	Sweden, Europe	SLE	(1) Antidepressants (N06AX)	Median 65, IQR 48–76	01/01/2006–31/12/2009	Case-control	Retrospective
Roberts 2018	US, North America	SLE	(1) SSRI (2) Tricyclic and other antidepressants	Range 28–93	Study I: 1996–2012, study II: 1993–2013	Cohort	Prospective
Schoonen 2010	UK, Europe	SLE	(1) Carbamazepine (2) Chlorpromazine	Range 4.5–88.4	1987–2001	Nested case-control	Retrospective
Mirza 2021	US, North America	Alopecia areata	Antidepressants	Range 30–55	2002–2012	Cohort	Prospective
Vallerand 2019	UK, Europe	Alopecia areata	Antidepressants	Range 10–90, median 36.6, IQR 24.0	1986–2012	Cohort	Retrospective
Brauchli 2009	UK, Europe	Psoriasis	(1) SSRI (2) Lithium (3) Atypical antipsychotics (4) Butyrophenones (5) Phenothiazines (6) Other typical antipsychotics (7) Olanzapine	No figures, assumably adults and elderly	01/01/1994–31/12/2005	Case-control	Retrospective
Tzeng 2021	Taiwan, Asia	Psoriasis	(1) Antidepressants (2) SSRI (3) TCAs (4) SNRI (5) Other antidepressants	20 years and older, mean 48.0 ± 16.7	1/2001–12/2010	Cohort	Retrospective
Sparks 2021	US, North America	Rheumatoid arthritis	Antidepressants	Range 25–55	Nurses’ Health Study, NHS 1992–2014, NHSII 1993–2015	Cohort	Prospective
Vallerand 2018	UK, Europe	Rheumatoid arthritis	Antidepressants	Range 10–90, median 36.6, IQR 24.0	1986–2012	Cohort	Retrospective
Garbe 2012	Germany, Europe	Immune thrombocytopenia	(1) Melperone (2) Promethazine	Outpatients: median 56, range 18–90. Inpatients: median 66, range 29–91	10/2000–03/2009	Case-control	Prospective
Rosenberg 2017	Canada, North America	Interstitial lung disease	SSRI and SNRI together	Range 75–93, cases mean 89.0, control mean 88.7	Patients active practice members 01/02/2016	Case-control	Retrospective
Nielsen 2015	Denmark, Europe	Multiple sclerosis	Valproic acid	18–54	1/1/1997–31/12/2011	Cohort	Retrospective
Miller 2001	US, North America	Silent thyreoiditis	Lithium	Range 24–49, mean 34 ± 6	1/1992–7/1996	Case-control	Retrospective
Vallerand 2019	UK, Europe	Vitiligo	Antidepressants	Range 10–90, median 36.6, IQR 24.0	1986–2012	Cohort	Retrospective

CC = Collagenous colitis

IQR = Interquartile range

LC = Lymphocytic colitis

MC = Microscopic colitis

NR = Not reported

SD = Standard deviation

**Fig 2:**
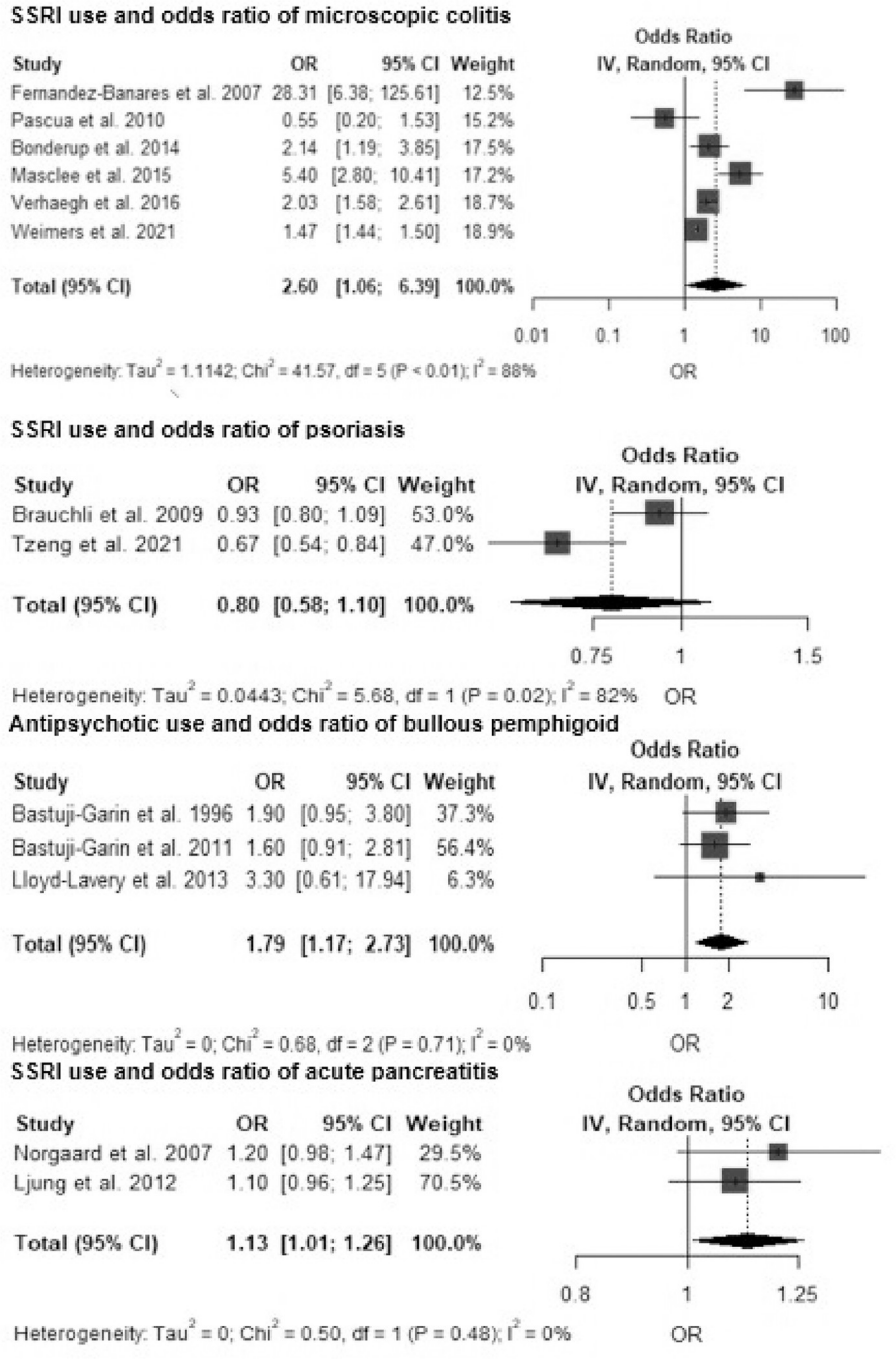
Forest plots for available meta-analyses of the risk of immune-mediated inflammatory diseases by the use of selective serotonin reuptake inhibitors (SSRI) and antipsychotics.

**Fig 3:**
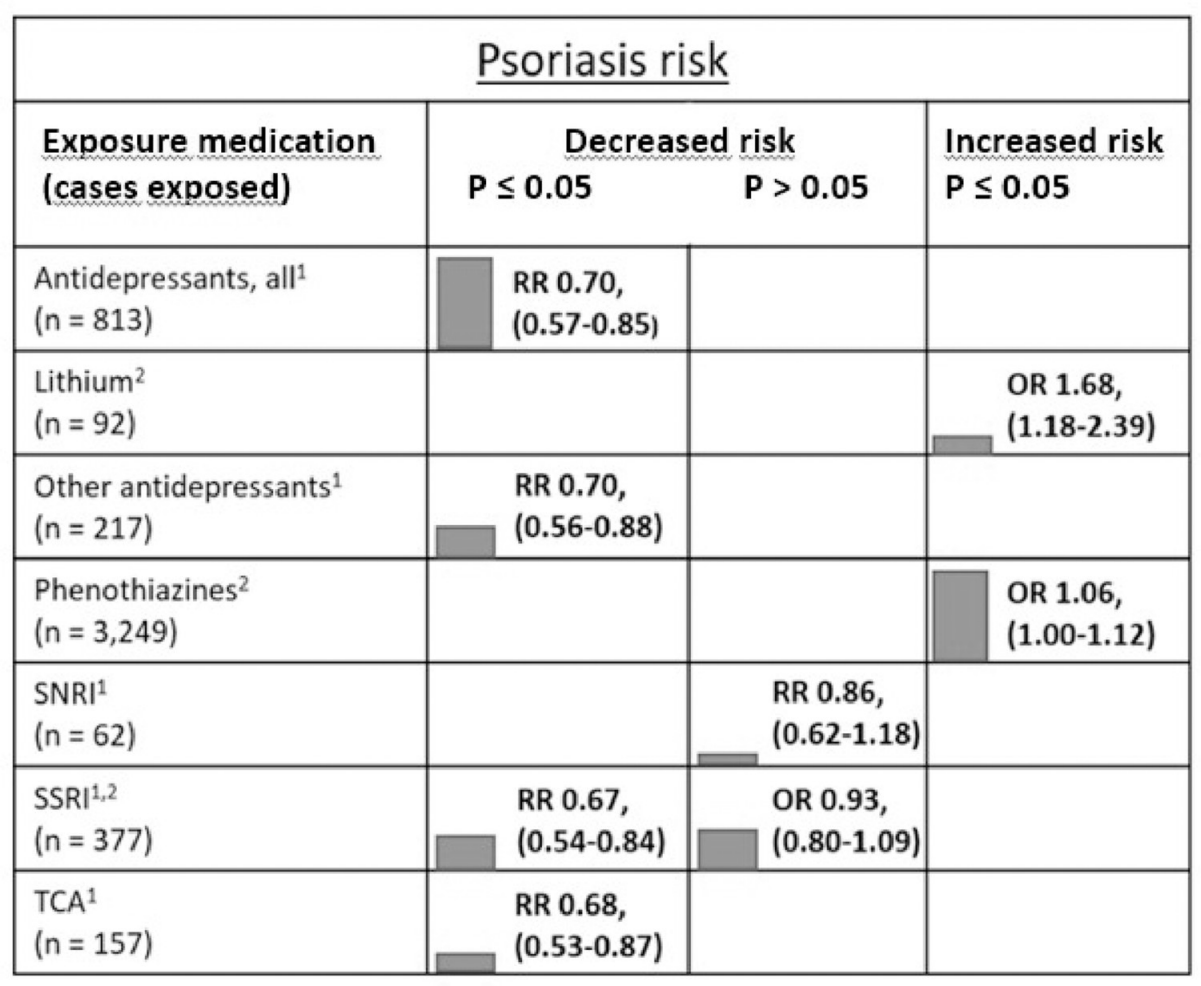
Harvest plot of the associations between the use of psychiatric medication and risk of psoriasis. ^1^ Tzeng et al. (2021) is a cohort study reporting relative risks (RRs). ^2^ Brauchli et al. (2009) is a case-control study with odds ratios (ORs) of the association shown. The harvest plot shows the risk of psoriasis by the type of psychiatric medication. Only associations with at least 50 psoriasis patients with the drug of interest are shown. The height of the column indicates the size of the study, and the green color represents a study with a low risk of bias according to the Newcastle–Ottawa Scale.

Because of the limited number of studies by exposure-outcome pairs, a meta-analysis was possible only for 1) antipsychotics (any type) and bullous pemphigoid, 2) SSRIs and MC (including both histological subtypes CC and LC), 3) SSRIs and psoriasis, and 4) SSRIs and acute pancreatitis (Fig 2). Other single associations with at least 50 patients with the diagnosis and the use of psychiatric medication are visualized in modified harvest plots (Fig 3). The limited number of exposure–outcome pairs also prevented the use of a funnel plot with Egger’s regression to investigate publication bias or any other meta-regression investigation of study-level variables [46]. Among the different methods to control for confounding in eight cohort studies, only one used propensity-score matching [42].

### Microscopic colitis and its subtypes

Seven case-control studies investigated the odds of MC (or CC and LC) by exposure to SSRIs (any) or sertraline [16]. The overall association between SSRIs and MC had a very high heterogeneity with an OR of 2.60 (95% CI 1.06–6.39, I^2^ 97.45%, 6 studies, at least 1,322 exposed cases). The studies by Fernández-Bañares and Pascua had the highest risk of bias (NOS scores 5 and 6, respectively) [17, 19]. In a sensitivity analysis of four studies with the lowest risk of bias, heterogeneity remained high while the meta-OR was only marginally lower, 2.26 (95% CI 1.37–3.73, I^2^ 92.01%, 4 studies) than in the analysis of all SSRI studies. All studies except Weimers et al. reported the numbers of individuals in the analysis [21].

A meta-analysis by histologic subtype of MC showed a statistically significant association between SSRIs and LC (meta-OR 2.88, 95% CI 2.60–3.18, I^2^ 00.00%, 3 studies) but not between SSRIs and CC (meta-OR 2.32, 95% CI 0.55–9.81, I^2^ 95.53%, 3 studies). Based on GRADE, the certainty of the evidence was low, with considerably high heterogeneity.

### Psoriasis

Psoriasis was investigated by Brauchli et al. in a case-control study based on the nationwide GPRD patient register in the UK and by Tzeng et al. in a cohort study based on the nationwide health insurance registry in Taiwan [37, 38]. Both studies were considered to have a low risk of bias. The Taiwanese study found a lower psoriasis risk among the users of any kind of antidepressants analyzed together, and also when analyzed by the main antidepressant classes (SNRI, SSRI, TCA) (Fig 3). The incidence of psoriasis was 2.80 (95% CI 2.61–3.00) per 1,000 person-years among users of any antidepressants and 3.82 (95% CI 3.15–4.59) per 1,000 person-years among non-users. A meta-analysis was possible for SSRI use and psoriasis risk, and the difference was not statistically significant (meta-OR 0.80, 95% CI 0.58–1.10, I^2^ 82.39%, 2 studies). In individual studies, only lithium and phenothiazines were associated with a slightly increased psoriasis risk (Fig 3). All associations for psoriasis were considered to be of low certainty based on GRADE.

### Bullous pemphigoid

Bullous pemphigoid as outcome was investigated in four case-control studies, all representing studies with a relatively higher risk of bias (NOS score range 5–6) [28–31]. Meta-analysis was possible for three studies with antipsychotics as the exposure (meta-OR 1.79, 95% CI 1.17–2.73, I^2^ 0%). However, these studies were small-scale, together contributing only 48 patients for the meta-analysis. The only reasonably powered study (Varpuluoma et al. 2019) was not included in the meta-analysis, as it reported individual medications instead of broader classes [31]. In the study by Varpuluoma, controls were not from a population-based sample; rather, they were patients with basal cell carcinoma. They reported the highest risk of bullous pemphigoid in relation to hydroxyzine (aOR 17.3, 95% CI 11.5–26.0), periciazine (aOR 7.55, 95% CI 2.91–10.6), melperone (aOR 4.0, 95% CI 2.46–6.49), and risperidone (aOR 3.04, 95% CI 2.38–3.89).

### Pancreatitis

AP was the outcome in six case-control studies, all representing a low risk of bias according to NOS (score 9 for all) [22–27]. For reasonably similar medication–outcome pairs, a meta-analysis was possible for only two studies on the current use of SSRIs and acute pancreatitis (meta-OR 1.13, 95% CI 1.01–1.26, I^2^ 00.00%, 649 SSRI-exposed patients) with adjustment for major confounding factors, including alcohol-related diseases. Current SNRI use was associated with a marginally increased pancreatitis risk after the adjustment of relevant confounders (aOR 1.2, 95% CI 1.0–1.4) [25]. The estimates for both SSRIs and SNRIs and the development of pancreatitis represent low-certainty evidence.

Gasse et al. were the only ones to investigate the potency of conventional antipsychotics in blocking dopamine receptors with pancreatitis as the outcome [23]. The current use of preparations with high potency in blocking dopamine receptors was not associated with a statistically significant pancreatitis risk (aOR 1.2, 95% CI 0.7–2.0), whereas the use of intermediate-potency preparations (aOR 1.5, 95% CI 1.0–2.2) and low-potency preparations (aOR 2.8, 95% CI 2.0–3.8) were associated nearly 1.5 and three-fold odds, respectively. Neither the current use of atypical antipsychotics (OR 0.6, 95% CI 0.3–1.1) nor the previous use of clozapine or olanzapine (Boden 2012) had a statistically significant association with pancreatitis.

The current use of valproic acid increased the risk of pancreatitis in an analysis adjusted for, among other factors, alcohol-related diseases and gallstone disease (aOR 1.9, 95% CI 1.1–7.0). However, this estimate was based on only 16 valproic acid-exposed pancreatitis patients and thus constitutes very low-certainty evidence.

### Other diseases

Roberts et al. was the only cohort study on the risk of systemic lupus erythematosus (SLE), with a high risk of bias (NOS 5) [33]. They found similarly increased risks of SLE with the use of SSRIs (HR 2.63, 95% CI 1.66–4.14) and other antidepressants (HR 2.85, 95% CI 1.53–5.31) in an analysis adjusted for BMI, smoking, and oral contraceptive and postmenopausal hormone use. Schoonen et al. investigated both SLE and SCLE in a case-control study with a low risk of bias (NOS 9) [34]. Carbamazepine use increased the odds (aOR 1.88, 95% CI 1.09–3.22). Moreover, in a subgroup analysis on the number of prescriptions, the risk increase was related to longer use of carbamazepine (i.e., those with seven or more prescriptions) (aOR 3.04, 95% CI 1.34–6.85). The risk of SCLE was studied by Grönhagen et al., but the analysis involved only eight cases exposed to any antidepressants (OR 1.2, 95% CI 0.5–2.6) [32].

Two cohort studies assessed the risks of alopecia areata after exposure to any antidepressants with contrasting results. Vallerand et al. found a protective effect (HR 0.57, 95% CI 0.53–0.62), and Mirza et al. a modestly increased risk (RR 1.67, 95% CI 1.15–2.44) with the use of antidepressants [35, 36]. Similarly, Vallerand et al. also found a lower risk of rheumatoid arthritis (HR 0.74, 95% CI 0.71–0.76) in antidepressant users, while another study by Sparks et al. found a small increase in risk (HR 0.74, 95% CI 1.16–1.63) [36, 39].

Single studies assessed the risk of interstitial lung disease after SSRI or SNRI use (OR 8.79, 95% CI 2.40–32.2, Rosenberg 2017), silent thyroiditis after lithium use (OR 4.7, 95% CI 1.3–17.1), multiple sclerosis after valproic acid use (HR 1.30, 95% CI 0.44–3.80, Nilsen 2015), and vitiligo after antidepressant use (HR 0.58, 95% CI 0.54–0.63, Vallerand 2019) [42–45].

## Discussion

We systematically searched an extensive list of ADs and IMIDs and evaluated whether the use of psychiatric medications can be considered a risk factor for their development. Published associations between any psychiatric medications and immune diseases were considered to represent low to very low certainty of evidence at best. This means that the available literature cannot reliably confirm or refute whether associations really exist, and the true effect sizes might be markedly different from the available estimates.

In a meta-analysis, we found that SSRIs are associated with a 2–3-fold increase in the odds of developing MC. Furthermore, in a subgroup analysis by histological type, this risk appeared to be related to the development of LC rather than CC. However, considering the large imprecision and overall uncertainty, these analyses represent low-certainty evidence, and the estimate is, therefore, likely to change with further research. Serotonin is an important neurotransmitter in the gut, and animal models have shown SSRIs to increase the release of serotonin in the gut (Minami 2003), and that serotonin is involved in the inflammatory reaction of experimental colitis (Ghia 2009). Although common adverse effects of SSRIs include stomach complaints such as diarrhea, it remains uncertain whether SSRIs could trigger chronic inflammation manifesting in diarrhea. Hundreds of medications have been associated with chronic diarrhea, mainly in case reports and uncontrolled case series [47]. In controlled observational studies, however, proton-pump inhibitors (especially lansoprazole) have been linked with moderate certainty of the development of a chronic inflammatory diarrhea disease, namely MC and its subtype CC [48].

There was a non-statistically significant suggestion in two case-control studies that SSRI use would associate with a lower risk of psoriasis. All of the available outcome diseases are rare, and even in hypothetical causal cases, the absolute number of new diseases would be low. For example, using the incidence figures by Tzeng et al. 2021 to illustrate the scale of these risk magnitudes, and assuming the incidences were true, would yield a number needed to treat of 980 (95% CI 656–1936) to prevent the development of one psoriasis case with any antidepressant treatment [38]. SSRIs have been shown to have some anti-inflammatory effects, and a case-control study from Sweden found that patients with psoriasis who used SSRIs had less often need for systemic treatments [49]. Although there are findings of SSRIs having anti-inflammatory effects, how this would translate into changes in both psoriasis severity and incidence requires more epidemiological research preferably with a cohort study design.

BP is the most common bullous autoimmune disease affecting the subepidermal layer of the skin and mainly affects elderly people. Our meta-analysis of three studies found a 1.8-fold increase in the odds of BP after exposure to antipsychotics, representing low certainty of evidence due to these studies’ relatively high risk of bias. Such a modest effect size could be explained by confounding and biases, and larger prospective studies would be needed before antipsychotics could be considered risk factors for BP. The case-control study by Varpuluoma et al. (2019) reported only statistically significant associations, therefore risking reporting bias [31]. They reported 21 psychiatric medications (and additionally seven benzodiazepines, which we did not include in this review) that increase the risk of BP. We consider it biologically implausible that so many psychiatric medications spanning different classes could cause BP. These associations, therefore, most likely reflect systematic bias due to the selection of basal cell carcinoma patients as controls instead of a random sample from the general population. Such distortion would be expected to occur with any exposure associated with a lower risk of basal cell carcinoma but not related to BP, thus appearing in a case-control design as an increase in the odds of BP [50].

### Limitations

The limitations of this study mostly reflect limitations in the available literature and especially how results have been reported. Although some individual studies reported the number of prescriptions, the literature was too heterogenous and scarce for a meta-analysis on dose dependence.

Patients with immune diseases generally have higher than expected rates of psychiatric disorders, that are often treated with medications [51]. In studies on the associations between psychiatric medications and immune diseases, protopathic bias would occur if the exposure medication was used to manage the symptoms due to the yet unidentified AD or IMID. Because of the highly somatic manifestation of identified target diseases, we consider in the case of psychiatric medications, the possibility of protopathic bias to be less important than other major sources of bias, although we had no lag-time analyses available to support this assumption. In pharmacoepidemiological studies, confounding by indication is generally an important source of bias. However, it is probably less of an issue with our research question, as psychiatric medications are not used to manage the target diseases.

Assessing drug exposure independently of outcome data using nationwide registers eliminates recall bias and diminishes the impact of selection bias. Other approaches would introduce more uncertainty. For example, Pascua et al. (2010) had only a modest inter-rater agreement (kappa statistic 0.50) when patient-reported medications were compared to those found in electronic databases [19].

## Conclusions

Currently, in the case of psychiatric medications, the entire literature of comparative studies represents, at best, low certainty of evidence linking them to any AD or IMID. We have reviewed important sources of bias in the field for future studies to be more reliable. Identified associations, particularly that of SSRIs and the risk of MC, should be investigated in prospective cohort studies.

## Supporting information

S1 File Search strategy, MEDLINE (Ovid). (RTF)

S2 File Search strategy, Scopus. (RTF)

S3 File PRISMA 2020 checklist. (RTF)

S4 File Newcastle-Ottawa scale assessment of case-control and cohort studies for risk of bias. (RTF)

S5 File Data used in meta-analyses. (RTF)

## Abbreviations

AD: Autoimmune disease
aOR: Adjusted odds ratio
AP: Acute pancreatitis
CC: Collagenous colitis
CI: Confidence interval
DDD: Defined daily dose
DIL: Drug-induced lupus
GRADE: Grading of Recommendations, Assessment, Development, and Evaluations
HR: Hazard ratio
IMID: Immune-mediated inflammatory disease
IQR: Interquartile range
LC: Lymphocytic colitis
MC: Microscopic colitis
NOS: Newcastle–Ottawa Scale
OR: Odds ratio
PRISMA: Preferred reporting items for systematic review and meta-analysis protocols
PROSPERO: The International Prospective Register of Systematic ReviewsRR Risk ratio
SD: Standard deviation
SLE: Systemic lupus erythematosus
SNRI: Serotonin and norepinephrine reuptake inhibitor
SSRI: Selective serotonin reuptake inhibitor
TCA: Tricyclic antidepressant

## References

[1] Rosenblum MD, Remedios KA, Abbas AK. Mechanisms of human autoimmunity. J Clin Invest. 2015;125(6):2228–33. doi: 10.1172/JCI78088 25893595 PMC4518692

[2] Lerner A, Patricia J, Torsten M. The World Incidence and Prevalence of Autoimmune Diseases is Increasing. Int J Celiac Dis. 2015;3.4:151–155. doi: 10.12691/ijcd-3-4-8

[3] Miehlke S et al. European guidelines on microscopic colitis: United European Gastroenterology and European Microscopic Colitis Group statements and recommendations. UEG Journal. 2021;22;9(1):13–37. doi: 10.1177/2050640620951905 33619914 PMC8259259

[4] Macer BJ, Prady SL, Mikocka-Walus A. Antidepressants in Inflammatory Bowel Disease: A Systematic Review. Inflamm Bowel Dis. 2017;23(4):534–550. doi: 10.1097/MIB.0000000000001059 28267046

[5] Borchers AT, Keen CL, Gershwin ME. Drug-induced lupus. Ann N Y Acad Sci. 2007;1108:166–182. doi: 10.1196/annals.1422.019 17893983

[6] Vayne C, Guéry EA, Rollin J, Baglo T, Petermann R, Gruel Y. Pathophysiology and Diagnosis of Drug-Induced Immune Thrombocytopenia. J Clin Med. 2020;9(7):2212. doi: 10.3390/jcm9072212 32668640 PMC7408966

[7] Dowlati Y, Herrmann N, Swardfager W, et al. A meta-analysis of cytokines in major depression. Biol Psychiatry. 2010;67(5):446–457. doi: 10.1016/j.biopsych.2009.09.033 20015486

[8] Rengasamy M, Marsland A, Spada M, Hsiung K, Kovats T, Price RB. A chicken and egg scenario in psychoneuroimmunology: Bidirectional mechanisms linking cytokines and depression. J Affect Disord Rep. 2021;6:100177. doi: 10.1016/j.jadr.2021.100177 35992016 PMC9387766

[9] Page MJ, McKenzie JE, Bossuyt PM, et al. The PRISMA 2020 statement: An updated guideline for reporting systematic reviews. PLoS Med. 2021;18(3):e1003583. doi: 10.1371/journal.pmed.1003583 33780438 PMC8007028

[10] Wells G SB, O’Connell D, Peterson J, Welch V, Losos M et al. The Newcastle-Ottawa Scale (NOS) for assessing the quality of non-randomised studies in meta-analyses. 3rd Symposium on Systematic Reviews: Beyond the Basics. 2000. http://www.ohri.ca/programs/clinical_epidemiology/oxford.asp. Accessed September 27, 2021.

[11] Balshem H, Helfand M, Schunemann HJ, et al. GRADE guidelines: 3. Rating the quality of evidence. J Clin Epidemiol 2011;64:401–406. doi: 10.1016/j.jclinepi.2010.07.015 21208779

[12] Higgins JP, Thompson SG. Quantifying heterogeneity in a meta-analysis. Stat Med 2002;15;21(11):1539–58. doi: 10.1002/sim.1186 12111919

[13] Higgins JPT, Thomas J, Chandler J, Cumpston M, Li T, Page MJ, et al. Cochrane Handbook for Systematic Reviews of Interventions version 6.1 (updated September 2020). Cochrane, 2020. Available from www.training.cochrane.org/handbook. Accessed September 27, 2021.

[14] Ogilvie D, Fayter D, Petticrew M, et al. The harvest plot: a method for synthesising evidence about the differential effects of interventions. BMC Med Res Methodol. 2008;8:8. doi: 10.1186/1471-2288-8-8 18298827 PMC2270283

[15] Bonderup OK, Fenger-Grøn M, Wigh T, Pedersen L, Nielsen GL. Drug exposure and risk of microscopic colitis: a nationwide Danish case-control study with 5751 cases. Inflamm Bowel Dis. 2014;20(10):1702–1707. doi: 10.1097/MIB.0000000000000143 25153503

[16] Fernández-Bañares F, de Sousa MR, Salas A, et al. Epidemiological risk factors in microscopic colitis: a prospective case-control study. Inflamm Bowel Dis. 2013;19(2):411–417. doi: 10.1002/ibd.23009 23344243

[17] Fernández-Bañares F, Esteve M, Espinós JC, et al. Drug consumption and the risk of microscopic colitis. Am J Gastroenterol. 2007;102(2):324–330. doi: 10.1111/j.1572-0241.2006.00902.x 17100977

[18] Masclee GM, Coloma PM, Kuipers EJ, Sturkenboom MC. Increased risk of microscopic colitis with use of proton pump inhibitors and non-steroidal anti-inflammatory drugs. Am J Gastroenterol. 2015;110(5):749–759. doi: 10.1038/ajg.2015.119 25916221

[19] Pascua MF, Kedia P, Weiner MG, Holmes J, Ellenberg J, Lewis JD. Microscopic colitis and Medication Use. Clin Med Insights Gastroenterol. 2010;2010(3):11–19. doi: 10.4137/cgast.s4469 20640056 PMC2903747

[20] Verhaegh BP, de Vries F, Masclee AA, et al. High risk of drug-induced microscopic colitis with concomitant use of NSAIDs and proton pump inhibitors. Aliment Pharmacol Ther. 2016;43(9):1004–1013. doi: 10.1111/apt.13583 26956016

[21] Weimers P, Vedel Ankersen D, Lophaven SN, et al. Microscopic Colitis in Denmark: Regional Variations in Risk Factors and Frequency of Endoscopic Procedures. J Crohns Colitis. 2021;16(1):49–56. doi: 10.1093/ecco-jcc/jjab119 34232280

[22] Bodén R, Bexelius TS, Mattsson F, Lagergren J, Lindblad M, Ljung R. Antidopaminergic drugs and acute pancreatitis: a population-based study. BMJ Open. 2012;2(3):e000914. Published 2012 May 11. doi: 10.1136/bmjopen-2012-000914 22581796 PMC3353129

[23] Gasse C, Jacobsen J, Pedersen L, et al. Risk of hospitalization for acute pancreatitis associated with conventional and atypical antipsychotics: a population-based case-control study. Pharmacotherapy. 2008;28(1):27–34. doi: 10.1592/phco.28.1.27 18154471

[24] Lin HF, Liao KF, Chang CM, Lin CL, Lai SW. Association of use of selective serotonin reuptake inhibitors with risk of acute pancreatitis: a case-control study in Taiwan. Eur J Clin Pharmacol. 2017;73(12):1615–1621. doi: 10.1007/s00228-017-2328-x 28856398

[25] Ljung R, Rück C, Mattsson F, Bexelius TS, Lagergren J, Lindblad M. Selective serotonin reuptake inhibitors and the risk of acute pancreatitis: a Swedish population-based case-control study. J Clin Psychopharmacol. 2012;32(3):336–340. doi: 10.1097/JCP.0b013e318253d71a 22544014

[26] Nørgaard M, Jacobsen J, Ratanajamit C, et al. Valproic acid and risk of acute pancreatitis: a population-based case-control study. Am J Ther. 2006;13(2):113–117. doi: 10.1097/00045391-200603000-00005 16645426

[27] Nørgaard M, Jacobsen J, Gasse C, Pedersen L, Mortensen PB, Sørensen HT. Selective serotonin reuptake inhibitors and risk of acute pancreatitis: a population-based case-control study. J Clin Psychopharmacol. 2007;27(3):259–262. doi: 10.1097/JCP.0b013e318058a9c3 17502772

[28] Bastuji-Garin S, Joly P, Picard-Dahan C, et al. Drugs associated with bullous pemphigoid. A case-control study. Arch Dermatol. 1996;132(3):272–276. doi: 10.1001/archderm.1996.03890270044006 8607630

[29] Bastuji-Garin S, Joly P, Lemordant P, et al. Risk factors for bullous pemphigoid in the elderly: a prospective case-control study. J Invest Dermatol. 2011;131(3):637–643. doi: 10.1038/jid.2010.301 20944650

[30] Lloyd-Lavery A, Chi CC, Wojnarowska F, Taghipour K. The associations between bullous pemphigoid and drug use: a UK case-control study. JAMA Dermatol. 2013;149(1):58–62. doi: 10.1001/2013.jamadermatol.376 23324757

[31] Varpuluoma O, Jokelainen J, Försti AK, et al. Drugs used for neurologic and psychiatric conditions increase the risk for bullous pemphigoid: A case-control study. J Am Acad Dermatol. 2019;81(1):250–253. doi: 10.1016/j.jaad.2019.02.017 30771421

[32] Grönhagen CM, Fored CM, Linder M, Granath F, Nyberg F. Subacute cutaneous lupus erythematosus and its association with drugs: a population-based matched case-control study of 234 patients in Sweden. Br J Dermatol. 2012;167(2):296–305. doi: 10.1111/j.1365-2133.2012.10969.x 22458771

[33] Roberts AL, Kubzansky LD, Malspeis S, Feldman CH, Costenbader KH. Association of Depression With Risk of Incident Systemic Lupus Erythematosus in Women Assessed Across 2 Decades. JAMA Psychiatry. 2018;75(12):1225–1233. doi: 10.1001/jamapsychiatry.2018.2462 30208373 PMC6583686

[34] Schoonen WM, Thomas SL, Somers EC, et al. Do selected drugs increase the risk of lupus? A matched case-control study. Br J Clin Pharmacol. 2010;70(4):588–596. doi: 10.1111/j.1365-2125.2010.03733.x 20840450 PMC2950993

[35] Mirza MA, Jung SJ, Sun W, Qureshi AA, Cho E. Association of depression and alopecia areata in women: A prospective study. J Dermatol. 2021;48(8):1296–1298. doi: 10.1111/1346-8138.15931 34128269

[36] Vallerand IA, Lewinson RT, Parsons LM, et al. Assessment of a Bidirectional Association Between Major Depressive Disorder and Alopecia Areata. JAMA Dermatol. 2019;155(4):475–479. doi: 10.1001/jamadermatol.2018.4398 30649133 PMC6459092

[37] Brauchli YB, Jick SS, Curtin F, Meier CR. Lithium, antipsychotics, and risk of psoriasis. J Clin Psychopharmacol. 2009;29(2):134–140. doi: 10.1097/JCP.0b013e31819a4b7c 19512974

[38] Tzeng YM, Li IH, Kao HH, et al. Protective Effects of Anti-depressants against the Subsequent Development of Psoriasis in Patients with Major Depressive Disorder: a Cohort Study. J Affect Disord. 2021;281:590–596. doi: 10.1016/j.jad.2020.11.110 33257042

[39] Sparks JA, Malspeis S, Hahn J, et al. Depression and Subsequent Risk for Incident Rheumatoid Arthritis Among Women. Arthritis Care Res (Hoboken). 2021;73(1):78–89. doi: 10.1002/acr.24441 32937012 PMC7775283

[40] Vallerand IA, Lewinson RT, Frolkis AD, et al. Depression as a risk factor for the development of rheumatoid arthritis: a population-based cohort study. RMD Open. 2018;4(2):e000670. Published 2018 Jul 11. doi: 10.1136/rmdopen-2018-000670 30018804 PMC6045711

[41] Garbe E, Andersohn F, Bronder E, et al. Drug-induced immune thrombocytopaenia: results from the Berlin Case-Control Surveillance Study. Eur J Clin Pharmacol. 2012;68(5):821–832. doi: 10.1007/s00228-011-1184-3 22187020

[42] Rosenberg T, Lattimer R, Montgomery P, Wiens C, Levy L. The relationship of SSRI and SNRI usage with interstitial lung disease and bronchiectasis in an elderly population: a case-control study. Clin Interv Aging. 2017;12:1977–1984. Published 2017 Nov 21. doi: 10.2147/CIA.S144263 29200837 PMC5702166

[43] Nielsen NM, Svanström H, Stenager E, et al. The use of valproic acid and multiple sclerosis. Pharmacoepidemiol Drug Saf. 2015;24(3):262–268. doi: 10.1002/pds.3692 25111895

[44] Miller KK, Daniels GH. Association between lithium use and thyrotoxicosis caused by silent thyroiditis. Clin Endocrinol (Oxf). 2001;55(4):501–508. doi: 10.1046/j.1365-2265.2001.01381.x 11678833

[45] Vallerand IA, Lewinson RT, Parsons LM, et al. Vitiligo and major depressive disorder: A bidirectional population-based cohort study. J Am Acad Dermatol. 2019;80(5):1371–1379. doi: 10.1016/j.jaad.2018.11.047 30528503

[46] Egger M, Davey Smith G, Schneider M, Minder C. Bias in meta-analysis detected by a simple, graphical test. BMJ. 1997;315(7109):629–634. doi: 10.1136/bmj.315.7109.629 9310563 PMC2127453

[47] Chassany O, Michaux A, Bergmann JF. Drug-induced diarrhoea. Drug Saf. 2000;22(1):53–72. doi: 10.2165/00002018-200022010-00005 10647976

[48] Nevalainen A, Nevalainen O. Autoimmune and immune-mediated inflammatory diseases after exposure to acid-suppressive medication: a systematic review and meta-analysis. Int J Risk Saf Med. 2022;10.3233/JRS-220012. doi: 10.3233/JRS-220012 36442213

[49] Thorslund K, Svensson T, Nordlind K, Ekbom A, Fored CM. Use of serotonin reuptake inhibitors in patients with psoriasis is associated with a decreased need for systemic psoriasis treatment: a population-based cohort study. J Intern Med. 2013;274(3):281–287. doi: 10.1111/joim.12093 23711088

[50] Grimes DA, Schulz KF. Compared to what? Finding controls for case-control studies. Lancet. 2005;365(9468):1429–1433. doi: 10.1016/S0140-6736(05)66379-9 15836892

[51] Marrie RA, Walld R, Bolton JM, et al. Increased incidence of psychiatric disorders in immune-mediated inflammatory disease. J Psychosom Res. 2017;101:17–23. doi: 10.1016/j.jpsychores.2017.07.015 28867419

