# Supplementary material for: Psychiatric medications and the risk of autoimmune and immune-mediated inflammatory diseases: A systematic review and meta-analysis of observational studies
Source: PLoS One. 2023 Feb 28;18(2):e0281979. doi: 10.1371/journal.pone.0281979 (PMC9974122; doi:10.1371/journal.pone.0281979)
Supplement: S1 File — (RTF) [file pone.0281979.s001.rtf]

S1 
Search strategy, MEDLINE (Ovid)
Search from the beginning of indexing in MEDLINE until 28.11.2021: 3838 documents
1.	carbamazepine.mp. OR exp Carbamazepine/
2.	oxcarbazepine.mp. OR exp Oxcarbazepine/
3.	Valproic acid.mp. OR exp Valproic Acid/
4.	valproate.mp. OR exp Valproic Acid/
5.	lamotrigine.mp. OR exp Lamotrigine/
6.	exp Antipsychotic Agents/ OR antipsychotic*.mp.
7.	lithium.mp. OR exp Lithium/
8.	exp Antidepressive Agents/ OR antidepressant*.mp.
9.	desipramine.mp. OR exp Desipramine/
10.	imipramine.mp. OR exp Imipramine/
11.	clomipramine.mp. OR exp Clomipramine/
12.	opipramol.mp. OR exp Opipramol/
13.	trimipramine.mp. OR exp Trimipramine/
14.	lofepramine.mp. OR exp Lofepramine/
15.	exp Dibenzazepines/ OR dibenzepin.mp.
16.	exp Amitriptyline/ OR amitriptyline.mp.
17.	Nortriptyline.mp. OR exp Nortriptyline/
18.	Protriptyline.mp. OR exp Protriptyline/
19.	Doxepin.mp. OR exp Doxepin/
20.	Iprindole.mp. OR exp Iprindole/
21.	Melitracen.mp.
22.	Butriptyline.mp.
23.	Dosulepin.mp. OR exp Dothiepin/
24.	Amoxapine.mp. OR exp Amoxapine/
25.	Dimetacrine.mp.
26.	Amineptine.mp.
27.	Maprotiline.mp. OR exp Maprotiline/
28.	Quinupramine.mp.
29.	exp Antidepressive Agents, Tricyclic/
30.	exp Serotonin Uptake Inhibitors/ OR SSRI.mp.
31.	Zimelidine.mp. OR exp Zimeldine/
32.	Fluoxetine.mp. OR exp Fluoxetine/
33.	Citalopram.mp. OR exp Citalopram/
34.	Paroxetine.mp. OR exp Paroxetine/
35.	Sertraline.mp. OR exp Sertraline/
36.	Alaproclate.mp.
37.	Fluvoxamine.mp. OR exp Fluvoxamine/
38.	Etoperidone.mp.
39.	Escitalopram.mp. OR exp Citalopram/
40.	Monoamine oxidase inhibitors.mp. OR exp Monoamine Oxidase Inhibitors/
41.	exp Isocarboxazid/ OR Isocarboxazid.mp.
42.	Nialamide.mp. OR exp Nialamide/
43.	Phenelzine.mp. OR exp Phenelzine/
44.	Tranylcypromine.mp. OR exp Tranylcypromine/
45.	exp Monoamine Oxidase Inhibitors/ OR Iproniazide.mp.
46.	Iproclozide.mp.
47.	Moclobemide.mp. OR exp Moclobemide/
48.	exp Monoamine Oxidase Inhibitors/ OR Toloxatone.mp.
49.	Oxitriptan.mp. OR exp 5-Hydroxytryptophan/
50.	Mianserin.mp. OR exp Mianserin/
51.	Nomifensine.mp. OR exp Nomifensine/
52.	exp Trazodone/ OR Trazodone.mp.
53.	Nefazodone.mp.
54.	Minaprine.mp.
55.	Bifemelane.mp.
56.	Viloxazine.mp. OR exp Viloxazine/
57.	Oxaflozane.mp.
58.	Mirtazapine.mp. OR exp Mirtazapine/
59.	Bupropion.mp. OR exp Bupropion/
60.	Medifoxamine.mp.
61.	Tianeptine.mp.
62.	Pivagabine.mp.
63.	Venlafaxine.mp. OR exp Venlafaxine Hydrochloride/
64.	Milnacipran.mp. OR exp Milnacipran/
65.	Reboxetine.mp. OR exp Reboxetine/
66.	Gepirone.mp.
67.	Duloxetine.mp. OR exp Duloxetine Hydrochloride/
68.	Agomelatine.mp.
69.	Desvenlafaxine.mp. OR exp Desvenlafaxine Succinate/
70.	Vilazodone.mp. OR exp Vilazodone Hydrochloride/
71.	Vortioxetine.mp. OR Vortioxetine/
72.	exp Chlorpromazine/ OR chlorpromazine.mp.
73.	haloperidol.mp. OR exp Haloperidol/
74.	perphenazine.mp. OR exp Perphenazine/
75.	fluphenazine.mp. OR exp Fluphenazine/
76.	risperidone.mp. OR exp Risperidone/
77.	olanzapine.mp. OR exp Olanzapine/
78.	quetiapine.mp. OR exp Quetiapine Fumarate/
79.	ziprasidone.mp.
80.	aripiprazole.mp. OR exp Aripiprazole/
81.	paliperidone.mp. OR exp Paliperidone Palmitate/
82.	lurasidone.mp. OR exp Lurasidone Hydrochloride/
83.	asenapine.mp.
84.	clozapine.mp. OR exp Clozapine/
85.	iloperidone.mp.
86.	levomepromazine.mp. OR exp Methotrimeprazine/
87.	perazine.mp. OR exp Perazine/
88.	zuclopenthixol.mp. OR exp Clopenthixol/
89.	1 OR 2 OR 3 OR 4 OR 5 OR 6 OR 7 OR 8 OR 9 OR 10 OR 11 OR 12 OR 13 OR 14 OR 15 OR 16 OR 17 OR 18 OR 19 OR 20 OR 21 OR 22 OR 23 OR 24 OR 25 OR 26 OR 27 OR 28  OR 29 OR 30 OR 31 OR 32 OR 33 OR 34 OR 35 OR 36 OR 37 OR 38 OR 39 OR 40 OR 41 OR 42 OR 43 OR 44 OR 45 OR 46 OR 47 OR 48 OR 49 OR 50 OR 51 OR 52 OR 53 OR 54 OR 55 OR 56 OR 57 OR 58 OR 59 OR 60 OR 61 OR 62 OR 63 OR 64 OR 65 OR 66 OR 67 OR 68 OR 69 OR 70 OR 71 OR 72 OR 73 OR 74 OR 75 OR 76 OR 77 OR 78 OR 79 OR 80 OR 81 OR 82 OR 83 OR 84 OR 85 OR 86 OR 87 OR 88
90.	Addison's Disease.mp. OR Addison Disease/
91.	(addison* adj1 disease*).mp. [mp=title, abstract, original title, name of substance word, subject heading word, floating sub-heading word, keyword heading word, organism supplementary concept word, protocol supplementary concept word, rare disease supplementary concept word, unique identifier, synonyms]
92.	(adren* adj1 (insufficienc* OR autoimmun*)).mp. [mp=title, abstract, original title, name of substance word, subject heading word, floating sub-heading word, keyword heading word, organism supplementary concept word, protocol supplementary concept word, rare disease supplementary concept word, unique identifier, synonyms]
93.	Alopecia Areata.mp. OR exp Alopecia Areata/
94.	(alopecia adj1 totalis).mp. [mp=title, abstract, original title, name of substance word, subject heading word, floating sub-heading word, keyword heading word, organism supplementary concept word, protocol supplementary concept word, rare disease supplementary concept word, unique identifier, synonyms]
95.	(alopecia adj1 universalis).mp. [mp=title, abstract, original title, name of substance word, subject heading word, floating sub-heading word, keyword heading word, organism supplementary concept word, protocol supplementary concept word, rare disease supplementary concept word, unique identifier, synonyms]
96.	Anemia, hemolytic, autoimmune.mp. OR Anemia, Hemolytic, Autoimmune/
97.	((anemia* OR anaemia* OR hemoly* OR haemoly*) adj2 autoimmun*).mp. [mp=title, abstract, original title, name of substance word, subject heading word, floating sub-heading word, keyword heading word, organism supplementary concept word, protocol supplementary concept word, rare disease supplementary concept word, unique identifier, synonyms]
98.	Anemia, Pernicious.mp. OR Anemia, Pernicious/
99.	((addison* OR pernicious) adj1 (anaemia OR anemia)).mp. [mp=title, abstract, original title, name of substance word, subject heading word, floating sub-heading word, keyword heading word, organism supplementary concept word, protocol supplementary concept word, rare disease supplementary concept word, unique identifier, synonyms]
100.	(autoimmun* adj1 gastriti*).mp. [mp=title, abstract, original title, name of substance word, subject heading word, floating sub-heading word, keyword heading word, organism supplementary concept word, protocol supplementary concept word, rare disease supplementary concept word, unique identifier, synonyms]
101.	Anti-Glomerular Basement Membrane Disease.mp. OR Anti-Glomerular Basement Membrane Disease/
102.	((goodpasture* OR anti-GBM OR GBM OR anti-glomerular OR anti glomerular) adj1 (disease or syndrome)).mp. [mp=title, abstract, original title, name of substance word, subject heading word, floating sub-heading word, keyword heading word, organism supplementary concept word, protocol supplementary concept word, rare disease supplementary concept word, unique identifier, synonyms]
103.	Anti-Neutrophil Cytoplasmic Antibody-Associated Vasculitis.mp. OR exp Anti-Neutrophil Cytoplasmic Antibody-Associated   Vasculitis/
104.	((ANCA OR pausi-immune) adj1 (associated OR vasculiti*)).mp. [mp=title, abstract, original title, name of substance word, subject heading word, floating sub-heading word, keyword heading word, organism supplementary concept word, protocol supplementary concept word, rare disease supplementary concept word, unique identifier, synonyms]
105.	Anti-N-Methyl-D-Aspartate Receptor Encephalitis.mp. OR Anti-N-Methyl-D-Aspartate Receptor Encephalitis/
106.	(NMDA* adj2 encephaliti*).mp. [mp=title, abstract, original title, name of substance word, subject heading word, floating sub-heading word, keyword heading word, organism supplementary concept word, protocol supplementary concept word, rare disease supplementary concept word, unique identifier, synonyms]
107.	Antibodies, Antineutrophil Cytoplasmic.mp. OR Antibodies, Antineutrophil Cytoplasmic/
108.	ANCA.mp. OR exp Antibodies, Antineutrophil Cytoplasmic/
109.	Antiphospholipid Syndrome.mp. OR Antiphospholipid Syndrome/
110.	((hughes OR antiphospholipid OR phospholipid OR anti-phospholipid) adj1 syndrome*).mp. [mp=title, abstract, original title, name of substance word, subject heading word, floating sub-heading word, keyword heading word, organism supplementary concept word, protocol supplementary concept word, rare disease supplementary concept word, unique identifier, synonyms]
111.	aortitis.mp. OR Aortitis/
112.	aortiti*.mp.
113.	arteritis.mp. OR Arteritis/
114.	arteriti*.mp.
115.	(arterial adj1 inflammation).mp. [mp=title, abstract, original title, name of substance word, subject heading word, floating sub-heading word, keyword heading word, organism supplementary concept word, protocol supplementary concept word, rare disease supplementary concept word, unique identifier, synonyms]
116.	Arthritis, Psoriatic.mp. OR Arthritis, Psoriatic/
117.	Arthritis, Rheumatoid.mp. OR Arthritis, Rheumatoid/
118.	Arthritis, Juvenile.mp. OR Arthritis, Juvenile/
119.	Rheumatic Fever.mp. OR Rheumatic Fever/
120.	((arthritides OR arthritis OR fever* OR polyarthritis OR polyarthritides OR polymyalgia OR inflammator*) adj1 (psoria* OR reactive OR rheumat*)).mp. [mp=title, abstract, original title, name of substance word, subject heading word, floating sub-heading word, keyword heading word, organism supplementary concept word, protocol supplementary concept word, rare disease supplementary concept word, unique identifier, synonyms]
121.	Arthus Reaction.mp. OR Arthus Reaction/
122.	(arthus adj1 (phenomenon OR reaction)).mp. [mp=title, abstract, original title, name of substance word, subject heading word, floating sub-heading word, keyword heading word, organism supplementary concept word, protocol supplementary concept word, rare disease supplementary concept word, unique identifier, synonyms]
123.	Autoantibodies/ OR autoantibod*.mp.
124.	Autoimmune Diseases/ OR Autoimmune diseases.mp.
125.	(autoimmun* adj1 (disease* OR disorder*)).mp. [mp=title, abstract, original title, name of substance word, subject heading word, floating sub-heading word, keyword heading word, organism supplementary concept word, protocol supplementary concept word, rare disease supplementary concept word, unique identifier, synonyms]
126.	Autoimmune diseases of the nervous system.mp. OR "Autoimmune Diseases of the Nervous System"/
127.	((autoimmun* OR immun*) adj2 (neurologic OR nervous)).mp. [mp=title, abstract, original title, name of substance word, subject heading word, floating sub-heading word, keyword heading word, organism supplementary concept word, protocol supplementary concept word, rare disease supplementary concept word, unique identifier, synonyms]
128.	Autoimmune Hypophysitis.mp. OR Autoimmune Hypophysitis/
129.	((autoimmun* OR lympho*) adj1 (hypophysiti* OR panhypophysiti* OR adenohypophysiti*)).mp. [mp=title, abstract, original title, name of substance word, subject heading word, floating sub-heading word, keyword heading word, organism supplementary concept word, protocol supplementary concept word, rare disease supplementary concept word, unique identifier, synonyms]
130.	Autoimmune Lymphoproliferative Syndrome.mp. OR Autoimmune Lymphoproliferative Syndrome/
131.	(Canale-Smith OR Canale Smith).mp. [mp=title, abstract, original title, name of substance word, subject heading word, floating sub-heading word, keyword heading word, organism supplementary concept word, protocol supplementary concept word, rare disease supplementary concept word, unique identifier, synonyms]
132.	Pancreatitis.mp. OR Pancreatitis/
133.	pancreatiti*.mp. [mp=title, abstract, original title, name of substance word, subject heading word, floating sub-heading word, keyword heading word, organism supplementary concept word, protocol supplementary concept word, rare disease supplementary concept word, unique identifier, synonyms]
134.	Autoimmunity.mp. OR Autoimmunity/
135.	(autoimmun* OR autoantibod* OR autoinflammat*).mp. [mp=title, abstract, original title, name of substance word, subject heading word, floating sub-heading word, keyword heading word, organism supplementary concept word, protocol supplementary concept word, rare disease supplementary concept word, unique identifier, synonyms]
136.	(autoimmun* adj2 (angioedema* OR edema* OR skin OR cutis OR cutaneous OR urticaria)).mp. [mp=title, abstract, original title, name of substance word, subject heading word, floating sub-heading word, keyword heading word, organism supplementary concept word, protocol supplementary concept word, rare disease supplementary concept word, unique identifier, synonyms]
137.	(autoimmun* adj2 (neutropenia OR lymphocytopenia OR lymphopenia)).mp. [mp=title, abstract, original title, name of substance word, subject heading word, floating sub-heading word, keyword heading word, organism supplementary concept word, protocol supplementary concept word, rare disease supplementary concept word, unique identifier, synonyms]
138.	(autoimmun* adj2 (myocarditis OR heart)).mp. [mp=title, abstract, original title, name of substance word, subject heading word, floating sub-heading word, keyword heading word, organism supplementary concept word, protocol supplementary concept word, rare disease supplementary concept word, unique identifier, synonyms]
139.	(autoimmun* adj2 (oophoritis OR orchitis or gonad*)).mp. [mp=title, abstract, original title, name of substance word, subject heading word, floating sub-heading word, keyword heading word, organism supplementary concept word, protocol supplementary concept word, rare disease supplementary concept word, unique identifier, synonyms]
140.	(autoimmun* adj2 (polyglandular OR polyendocrinopath*)).mp. [mp=title, abstract, original title, name of substance word, subject heading word, floating sub-heading word, keyword heading word, organism supplementary concept word, protocol supplementary concept word, rare disease supplementary concept word, unique identifier, synonyms]
141.	(autoimmun* adj2 (retinopathy OR uveitis OR eye)).mp. [mp=title, abstract, original title, name of substance word, subject heading word, floating sub-heading word, keyword heading word, organism supplementary concept word, protocol supplementary concept word, rare disease supplementary concept word, unique identifier, synonyms]
142.	Behcet Syndrome.mp. OR Behcet Syndrome/
143.	(behcet* adj1 (syndrome OR disease*)).mp. [mp=title, abstract, original title, name of substance word, subject heading word, floating sub-heading word, keyword heading word, organism supplementary concept word, protocol supplementary concept word, rare disease supplementary concept word, unique identifier, synonyms]
144.	(triple symptom adj2 compl*).mp. [mp=title, abstract, original title, name of substance word, subject heading word, floating sub-heading word, keyword heading word, organism supplementary concept word, protocol supplementary concept word, rare disease supplementary concept word, unique identifier, synonyms]
145.	Celiac Disease.mp. OR Celiac Disease/
146.	((celiac OR coeliac OR gluten*) adj1 (enteropath* OR disease OR sprue)).mp. [mp=title, abstract, original title, name of substance word, subject heading word, floating sub-heading word, keyword heading word, organism supplementary concept word, protocol supplementary concept word, rare disease supplementary concept word, unique identifier, synonyms]
147.	Cholangitis, Sclerosing.mp. OR Cholangitis, Sclerosing/
148.	(sclerosing adj1 cholangiti*).mp. [mp=title, abstract, original title, name of substance word, subject heading word, floating sub-heading word, keyword heading word, organism supplementary concept word, protocol supplementary concept word, rare disease supplementary concept word, unique identifier, synonyms]
149.	Churg-Strauss Syndrome.mp. OR Churg-Strauss Syndrome/
150.	(eosinophilic adj2 granulomato* adj2 polyangiiti*).mp. [mp=title, abstract, original title, name of substance word, subject heading word, floating sub-heading word, keyword heading word, organism supplementary concept word, protocol supplementary concept word, rare disease supplementary concept word, unique identifier, synonyms]
151.	(churg adj1 strauss).mp. [mp=title, abstract, original title, name of substance word, subject heading word, floating sub-heading word, keyword heading word, organism supplementary concept word, protocol supplementary concept word, rare disease supplementary concept word, unique identifier, synonyms]
152.	Cogan Syndrome.mp. OR Cogan Syndrome/
153.	(syndrome adj1 cogan*).mp. [mp=title, abstract, original title, name of substance word, subject heading word, floating sub-heading word, keyword heading word, organism supplementary concept word, protocol supplementary concept word, rare disease supplementary concept word, unique identifier, synonyms]
154.	Colitis, Microscopic.mp. OR Colitis, Microscopic/
155.	((collagenous OR lymphocytic OR microscopic) adj1 colitis).mp. [mp=title, abstract, original title, name of substance word, subject heading word, floating sub-heading word, keyword heading word, organism supplementary concept word, protocol supplementary concept word, rare disease supplementary concept word, unique identifier, synonyms]
156.	Colitis, Ulcerative.mp. OR Colitis, Ulcerative/
157.	((ulcerative OR gravis) adj1 colitis).mp. [mp=title, abstract, original title, name of substance word, subject heading word, floating sub-heading word, keyword heading word, organism supplementary concept word, protocol supplementary concept word, rare disease supplementary concept word, unique identifier, synonyms]
158.	(inflammat* adj1 bowel).mp. [mp=title, abstract, original title, name of substance word, subject heading word, floating sub-heading word, keyword heading word, organism supplementary concept word, protocol supplementary concept word, rare disease supplementary concept word, unique identifier, synonyms]
159.	(granulomatous adj1 (colitis or enteritis)).mp. [mp=title, abstract, original title, name of substance word, subject heading word, floating sub-heading word, keyword heading word, organism supplementary concept word, protocol supplementary concept word, rare disease supplementary concept word, unique identifier, synonyms]
160.	idiopathic proctocolitis.mp. OR Colitis, Ulcerative/
161.	Inflammatory Bowel Diseases/ OR ibd.mp.
162.	Colitis, Ulcerative/ OR uc.mp.
163.	Connective tissue diseases.mp. OR Connective Tissue Diseases/
164.	(connective adj1 tissue adj1 (disease* OR disorder*)).mp. [mp=title, abstract, original title, name of substance word, subject heading word, floating sub-heading word, keyword heading word, organism supplementary concept word, protocol supplementary concept word, rare disease supplementary concept word, unique identifier, synonyms]
165.	CREST Syndrome.mp. OR CREST Syndrome/
166.	((CRST OR CREST) adj1 syndrom*).mp. [mp=title, abstract, original title, name of substance word, subject heading word, floating sub-heading word, keyword heading word, organism supplementary concept word, protocol supplementary concept word, rare disease supplementary concept word, unique identifier, synonyms]
167.	Crohn Disease.mp. OR Crohn Disease/
168.	((crohn* OR granulomatous) adj1 (disease* OR enterocolitis OR colitis OR enteritis)).mp. [mp=title, abstract, original title, name of substance word, subject heading word, floating sub-heading word, keyword heading word, organism supplementary concept word, protocol supplementary concept word, rare disease supplementary concept word, unique identifier, synonyms]
169.	ileocolitis.mp.
170.	(inflammat* adj2 bowel).mp. [mp=title, abstract, original title, name of substance word, subject heading word, floating sub-heading word, keyword heading word, organism supplementary concept word, protocol supplementary concept word, rare disease supplementary concept word, unique identifier, synonyms]
171.	((terminal OR regional) adj1 ileiti*).mp. [mp=title, abstract, original title, name of substance word, subject heading word, floating sub-heading word, keyword heading word, organism supplementary concept word, protocol supplementary concept word, rare disease supplementary concept word, unique identifier, synonyms]
172.	Demyelinating Autoimmune Diseases, CNS.mp. OR Demyelinating Autoimmune Diseases, CNS/
173.	(demyelinating adj3 autoimmune adj3 (CNS OR brain OR cerebral OR spinal OR central nervous)).mp. [mp=title, abstract, original title, name of substance word, subject heading word, floating sub-heading word, keyword heading word, organism supplementary concept word, protocol supplementary concept word, rare disease supplementary concept word, unique identifier, synonyms]
174.	Dermatitis Herpetiformis.mp. OR Dermatitis Herpetiformis/
175.	(duhring* adj1 disease).mp. [mp=title, abstract, original title, name of substance word, subject heading word, floating sub-heading word, keyword heading word, organism supplementary concept word, protocol supplementary concept word, rare disease supplementary concept word, unique identifier, synonyms]
176.	Dermatomyositis.mp. OR Dermatomyositis/
177.	dermatopolymyositis.mp. OR Dermatomyositis/
178.	((myositis OR dermatomyositis) adj1 (juvenile OR polymyositis* OR childhood)).mp. [mp=title, abstract, original title, name of substance word, subject heading word, floating sub-heading word, keyword heading word, organism supplementary concept word, protocol supplementary concept word, rare disease supplementary concept word, unique identifier, synonyms]
179.	Diabetes Mellitus, Type 1/ OR Diabetes Mellitus, Type 1.mp.
180.	((autoimmun* OR type 1) adj2 diabetes).mp. [mp=title, abstract, original title, name of substance word, subject heading word, floating sub-heading word, keyword heading word, organism supplementary concept word, protocol supplementary concept word, rare disease supplementary concept word, unique identifier, synonyms]
181.	insulin-dependent Diabetes.mp.
182.	Encephalomyelitis, Acute Disseminated.mp. OR Encephalomyelitis, Acute Disseminated/
183.	((disseminated OR autoimmun*) adj2 encephalomyeliti*).mp. [mp=title, abstract, original title, name of substance word, subject heading word, floating sub-heading word, keyword heading word, organism supplementary concept word, protocol supplementary concept word, rare disease supplementary concept word, unique identifier, synonyms]
184.	Endarteritis.mp. OR Endarteritis/
185.	endarteriti*.mp.
186.	Felty syndrome.mp. OR Felty Syndrome/
187.	(felt* adj1 syndrome).mp. [mp=title, abstract, original title, name of substance word, subject heading word, floating sub-heading word, keyword heading word, organism supplementary concept word, protocol supplementary concept word, rare disease supplementary concept word, unique identifier, synonyms]
188.	Giant Cell Arteritis.mp. OR Giant Cell Arteritis/
189.	((arteriti* OR aortiti*) adj1 (giant cell or temporal or cranial)).mp. [mp=title, abstract, original title, name of substance word, subject heading word, floating sub-heading word, keyword heading word, organism supplementary concept word, protocol supplementary concept word, rare disease supplementary concept word, unique identifier, synonyms]
190.	(horton* adj1 disease).mp. [mp=title, abstract, original title, name of substance word, subject heading word, floating sub-heading word, keyword heading word, organism supplementary concept word, protocol supplementary concept word, rare disease supplementary concept word, unique identifier, synonyms]
191.	Glomerulonephritis, IGA.mp. OR Glomerulonephritis, IGA/
192.	((glomerulonephriti* OR kindney) adj2 autoimmun*).mp. [mp=title, abstract, original title, name of substance word, subject heading word, floating sub-heading word, keyword heading word, organism supplementary concept word, protocol supplementary concept word, rare disease supplementary concept word, unique identifier, synonyms]
193.	Glomerulonephritis, Membranous.mp. OR Glomerulonephritis, Membranous/
194.	((immunog* OR membranous) adj1 (nephropathy OR glomerulonephriti*)).mp. [mp=title, abstract, original title, name of substance word, subject heading word, floating sub-heading word, keyword heading word, organism supplementary concept word, protocol supplementary concept word, rare disease supplementary concept word, unique identifier, synonyms]
195.	(heymann adj1 nephritis).mp. [mp=title, abstract, original title, name of substance word, subject heading word, floating sub-heading word, keyword heading word, organism supplementary concept word, protocol supplementary concept word, rare disease supplementary concept word, unique identifier, synonyms]
196.	Granulomatosis with Polyangiitis.mp. OR Granulomatosis with Polyangiitis/
197.	(granulomatosis adj1 polyangiiti*).mp. [mp=title, abstract, original title, name of substance word, subject heading word, floating sub-heading word, keyword heading word, organism supplementary concept word, protocol supplementary concept word, rare disease supplementary concept word, unique identifier, synonyms]
198.	(wegener* adj1 (granuloma* OR polyang*)).mp. [mp=title, abstract, original title, name of substance word, subject heading word, floating sub-heading word, keyword heading word, organism supplementary concept word, protocol supplementary concept word, rare disease supplementary concept word, unique identifier, synonyms]
199.	Graves Disease.mp. OR Graves Disease/
200.	((basedow* OR grave*) adj1 disease).mp. [mp=title, abstract, original title, name of substance word, subject heading word, floating sub-heading word, keyword heading word, organism supplementary concept word, protocol supplementary concept word, rare disease supplementary concept word, unique identifier, synonyms]
201.	Guillain-Barre Syndrome.mp. OR Guillain-Barre Syndrome/
202.	(guillain adj1 barre).mp. [mp=title, abstract, original title, name of substance word, subject heading word, floating sub-heading word, keyword heading word, organism supplementary concept word, protocol supplementary concept word, rare disease supplementary concept word, unique identifier, synonyms]
203.	(inflammatory adj1 (polyneuropath* OR polyradiculoneuropath*)).mp. [mp=title, abstract, original title, name of substance word, subject heading word, floating sub-heading word, keyword heading word, organism supplementary concept word, protocol supplementary concept word, rare disease supplementary concept word, unique identifier, synonyms]
204.	Hashimoto Disease.mp. OR Hashimoto Disease/
205.	(hashimoto* adj1 (disease OR struma OR thyroiditi*)).mp. [mp=title, abstract, original title, name of substance word, subject heading word, floating sub-heading word, keyword heading word, organism supplementary concept word, protocol supplementary concept word, rare disease supplementary concept word, unique identifier, synonyms]
206.	Hepatitis, Autoimmune.mp. OR Hepatitis, Autoimmune/
207.	(autoimmun* adj1 hepatiti*).mp. [mp=title, abstract, original title, name of substance word, subject heading word, floating sub-heading word, keyword heading word, organism supplementary concept word, protocol supplementary concept word, rare disease supplementary concept word, unique identifier, synonyms]
208.	Hypersomnolence, Idiopathic.mp. OR Idiopathic Hypersomnia/
209.	(hypersomn* adj1 idiopathic).mp. [mp=title, abstract, original title, name of substance word, subject heading word, floating sub-heading word, keyword heading word, organism supplementary concept word, protocol supplementary concept word, rare disease supplementary concept word, unique identifier, synonyms]
210.	Immunoglobulin G4-Related Disease.mp. OR Immunoglobulin G4-Related Disease/
211.	((IgG4-related OR immunoglobulin G4* OR IgG4*) adj2 (disease OR syndrome)).mp. [mp=title, abstract, original title, name of substance word, subject heading word, floating sub-heading word, keyword heading word, organism supplementary concept word, protocol supplementary concept word, rare disease supplementary concept word, unique identifier, synonyms]
212.	Lambert-Eaton myasthenic syndrome.mp. OR Lambert-Eaton Myasthenic Syndrome/
213.	(lambert* adj1 eaton).mp. [mp=title, abstract, original title, name of substance word, subject heading word, floating sub-heading word, keyword heading word, organism supplementary concept word, protocol supplementary concept word, rare disease supplementary concept word, unique identifier, synonyms]
214.	Latent Autoimmune Diabetes in Adults.mp. OR Latent Autoimmune Diabetes in Adults/
215.	LADA.mp.
216.	(latent adj2 diabetes).mp. [mp=title, abstract, original title, name of substance word, subject heading word, floating sub-heading word, keyword heading word, organism supplementary concept word, protocol supplementary concept word, rare disease supplementary concept word, unique identifier, synonyms]
217.	Lichen Planus.mp. OR Lichen Planus/
218.	(lichen adj2 planus).mp. [mp=title, abstract, original title, name of substance word, subject heading word, floating sub-heading word, keyword heading word, organism supplementary concept word, protocol supplementary concept word, rare disease supplementary concept word, unique identifier, synonyms]
219.	Lichenoid Eruptions.mp. OR Lichenoid Eruptions/
220.	(lichen* adj1 eruption*).mp. [mp=title, abstract, original title, name of substance word, subject heading word, floating sub-heading word, keyword heading word, organism supplementary concept word, protocol supplementary concept word, rare disease supplementary concept word, unique identifier, synonyms]
221.	Linear IgA Bullous Dermatosis.mp. OR Linear IgA Bullous Dermatosis/
222.	(dermatos* adj2 linear adj2 IgA).mp. [mp=title, abstract, original title, name of substance word, subject heading word, floating sub-heading word, keyword heading word, organism supplementary concept word, protocol supplementary concept word, rare disease supplementary concept word, unique identifier, synonyms]
223.	Liver Cirrhosis, Biliary.mp. OR Liver Cirrhosis, Biliary/
224.	(biliary adj1 (cirrhosis OR cirrhoses)).mp. [mp=title, abstract, original title, name of substance word, subject heading word, floating sub-heading word, keyword heading word, organism supplementary concept word, protocol supplementary concept word, rare disease supplementary concept word, unique identifier, synonyms]
225.	pbc.mp.
226.	Lupus Erythematosus, Systemic.mp. OR Lupus Erythematosus, Systemic/
227.	(lupus adj1 (cutaneous OR discoid OR erythematosus OR nephritis OR skin OR vasculitis)).mp. [mp=title, abstract, original title, name of substance word, subject heading word, floating sub-heading word, keyword heading word, organism supplementary concept word, protocol supplementary concept word, rare disease supplementary concept word, unique identifier, synonyms]
228.	(libman adj sacks).mp. [mp=title, abstract, original title, name of substance word, subject heading word, floating sub-heading word, keyword heading word, organism supplementary concept word, protocol supplementary concept word, rare disease supplementary concept word, unique identifier, synonyms]
229.	Lupus Nephritis.mp. OR Lupus Nephritis/
230.	(lupus adj1 (glomerulonephriti* OR nephriti*)).mp. [mp=title, abstract, original title, name of substance word, subject heading word, floating sub-heading word, keyword heading word, organism supplementary concept word, protocol supplementary concept word, rare disease supplementary concept word, unique identifier, synonyms]
231.	Lupus Vasculitis, Central Nervous System.mp. OR Lupus Vasculitis, Central Nervous System/
232.	((central nervous system OR CNS OR meningoencephaliti*) adj2 lupus).mp. [mp=title, abstract, original title, name of substance word, subject heading word, floating sub-heading word, keyword heading word, organism supplementary concept word, protocol supplementary concept word, rare disease supplementary concept word, unique identifier, synonyms]
233.	Malignant Atrophic Papulosis.mp. OR Malignant Atrophic Papulosis/
234.	((atrophic OR degos* OR kohlmeier*) adj1 (papuloses OR disease)).mp. [mp=title, abstract, original title, name of substance word, subject heading word, floating sub-heading word, keyword heading word, organism supplementary concept word, protocol supplementary concept word, rare disease supplementary concept word, unique identifier, synonyms]
235.	Mastocytosis/ OR Mastocytosis.mp.
236.	mastocytos*.mp.
237.	(mast cell adj2 (syndrome OR disease*)).mp. [mp=title, abstract, original title, name of substance word, subject heading word, floating sub-heading word, keyword heading word, organism supplementary concept word, protocol supplementary concept word, rare disease supplementary concept word, unique identifier, synonyms]
238.	Microscopic Polyangiitis.mp. OR Microscopic Polyangiitis/
239.	Mucocutaneous Lymph Node Syndrome.mp. OR Mucocutaneous Lymph Node Syndrome/
240.	((kawasaki OR mucocutaneous lymph node) adj1 (disease* OR syndrome*)).mp. [mp=title, abstract, original title, name of substance word, subject heading word, floating sub-heading word, keyword heading word, organism supplementary concept word, protocol supplementary concept word, rare disease supplementary concept word, unique identifier, synonyms]
241.	Multiple Sclerosis.mp. OR Multiple Sclerosis/
242.	((multiple OR disseminated) adj1 (sclerosis OR myelitis)).mp. [mp=title, abstract, original title, name of substance word, subject heading word, floating sub-heading word, keyword heading word, organism supplementary concept word, protocol supplementary concept word, rare disease supplementary concept word, unique identifier, synonyms]
243.	Myasthenia Gravis.mp. OR Myasthenia Gravis/
244.	(myastheni* adj1 (gravis OR disease OR syndrome)).mp. [mp=title, abstract, original title, name of substance word, subject heading word, floating sub-heading word, keyword heading word, organism supplementary concept word, protocol supplementary concept word, rare disease supplementary concept word, unique identifier, synonyms]
245.	myositi*.mp. OR Myositis/
246.	Narcolepsy.mp. OR Narcolepsy/
247.	((narcoleptic OR narcolepsy-cataplexy OR gelineau*) adj1 syndrom*).mp. [mp=title, abstract, original title, name of substance word, subject heading word, floating sub-heading word, keyword heading word, organism supplementary concept word, protocol supplementary concept word, rare disease supplementary concept word, unique identifier, synonyms]
248.	(paroxysmal adj1 sleep).mp. [mp=title, abstract, original title, name of substance word, subject heading word, floating sub-heading word, keyword heading word, organism supplementary concept word, protocol supplementary concept word, rare disease supplementary concept word, unique identifier, synonyms]
249.	Opsoclonus-Myoclonus Syndrome.mp. OR Opsoclonus-Myoclonus Syndrome/
250.	((opsoclonus* OR encephalopath*) adj1 myoclon*).mp. [mp=title, abstract, original title, name of substance word, subject heading word, floating sub-heading word, keyword heading word, organism supplementary concept word, protocol supplementary concept word, rare disease supplementary concept word, unique identifier, synonyms]
251.	(dancing adj1 (eyes OR feet)).mp. [mp=title, abstract, original title, name of substance word, subject heading word, floating sub-heading word, keyword heading word, organism supplementary concept word, protocol supplementary concept word, rare disease supplementary concept word, unique identifier, synonyms]
252.	Pemphigoid, Bullous.mp. OR Pemphigoid, Bullous/
253.	pemphigoid*.mp.
254.	Pemphigus/ OR Pemphigus.mp.
255.	(pemphigus adj1 (foliaceus OR vulgaris)).mp. [mp=title, abstract, original title, name of substance word, subject heading word, floating sub-heading word, keyword heading word, organism supplementary concept word, protocol supplementary concept word, rare disease supplementary concept word, unique identifier, synonyms]
256.	Phlebitis.mp. OR Phlebitis/
257.	(phlebiti* OR periphlebiti*).mp. [mp=title, abstract, original title, name of substance word, subject heading word, floating sub-heading word, keyword heading word, organism supplementary concept word, protocol supplementary concept word, rare disease supplementary concept word, unique identifier, synonyms]
258.	POEMS Syndrome.mp. OR POEMS Syndrome/
259.	(POEMS OR takatsuki* OR Crow Fukase OR Crow-Fukase).mp. [mp=title, abstract, original title, name of substance word, subject heading word, floating sub-heading word, keyword heading word, organism supplementary concept word, protocol supplementary concept word, rare disease supplementary concept word, unique identifier, synonyms]
260.	(polyneuropathy adj1 organomegal*).mp. [mp=title, abstract, original title, name of substance word, subject heading word, floating sub-heading word, keyword heading word, organism supplementary concept word, protocol supplementary concept word, rare disease supplementary concept word, unique identifier, synonyms]
261.	Polyarteritis Nodosa.mp. OR Polyarteritis Nodosa/
262.	((periarteritis OR polyarteriti* OR arteriti*) adj1 (nodosa OR essential OR necrotizing)).mp. [mp=title, abstract, original title, name of substance word, subject heading word, floating sub-heading word, keyword heading word, organism supplementary concept word, protocol supplementary concept word, rare disease supplementary concept word, unique identifier, synonyms]
263.	Polyendocrinopathies, Autoimmune.mp. OR Polyendocrinopathies, Autoimmune/
264.	(autoimmun* adj3 (polyendocrinopath* OR polyglandular OR oolyendocrine)).mp. [mp=title, abstract, original title, name of substance word, subject heading word, floating sub-heading word, keyword heading word, organism supplementary concept word, protocol supplementary concept word, rare disease supplementary concept word, unique identifier, synonyms]
265.	(AIRE adj1 deficienc*).mp. [mp=title, abstract, original title, name of substance word, subject heading word, floating sub-heading word, keyword heading word, organism supplementary concept word, protocol supplementary concept word, rare disease supplementary concept word, unique identifier, synonyms]
266.	(schmidt* adj1 syndrome).mp. [mp=title, abstract, original title, name of substance word, subject heading word, floating sub-heading word, keyword heading word, organism supplementary concept word, protocol supplementary concept word, rare disease supplementary concept word, unique identifier, synonyms]
267.	Polymyalgia Rheumatica.mp. OR Polymyalgia Rheumatica/
268.	(forestier* adj1 certonciny).mp. [mp=title, abstract, original title, name of substance word, subject heading word, floating sub-heading word, keyword heading word, organism supplementary concept word, protocol supplementary concept word, rare disease supplementary concept word, unique identifier, synonyms]
269.	(rheumatism adj1 peri*).mp. [mp=title, abstract, original title, name of substance word, subject heading word, floating sub-heading word, keyword heading word, organism supplementary concept word, protocol supplementary concept word, rare disease supplementary concept word, unique identifier, synonyms]
270.	((pseudopolyarthriti* OR pseudopolyarthriti*) adj1 rhizomelic).mp. [mp=title, abstract, original title, name of substance word, subject heading word, floating sub-heading word, keyword heading word, organism supplementary concept word, protocol supplementary concept word, rare disease supplementary concept word, unique identifier, synonyms]
271.	Polymyositis/ OR polymyositi*.mp.
272.	((multiple OR idiopathic OR ossificans) adj1 (myositi* OR polymyositi*)).mp. [mp=title, abstract, original title, name of substance word, subject heading word, floating sub-heading word, keyword heading word, organism supplementary concept word, protocol supplementary concept word, rare disease supplementary concept word, unique identifier, synonyms]
273.	Polyradiculoneuropathy, Chronic Inflammatory Demyelinating.mp. or Polyradiculoneuropathy, Chronic Inflammatory Demyelinating/
274.	CIDP.mp.
275.	((polyneuropath* OR polyradiculopath* OR polyradiculoneuropath*) adj2 inflammat*).mp. [mp=title, abstract, original title, name of substance word, subject heading word, floating sub-heading word, keyword heading word, organism supplementary concept word, protocol supplementary concept word, rare disease supplementary concept word, unique identifier, synonyms]
276.	Psoriasis.mp. OR Psoriasis/
277.	(psoria* OR (pustul* adj1 palm*)).mp. [mp=title, abstract, original title, name of substance word, subject heading word, floating sub-heading word, keyword heading word, organism supplementary concept word, protocol supplementary concept word, rare disease supplementary concept word, unique identifier, synonyms]
278.	Purpura, Schoenlein-Henoch.mp. OR Purpura, Schoenlein-Henoch/
279.	((henoch OR schoenlein OR anaphylactoid OR allergic OR rheumatoid OR hemorrhagica OR nonthrombo*) adj2 purpura*).mp. [mp=title, abstract, original title, name of substance word, subject heading word, floating sub-heading word, keyword heading word, organism supplementary concept word, protocol supplementary concept word, rare disease supplementary concept word, unique identifier, synonyms]
280.	Purpura, Thrombocytopenic, Idiopathic.mp. OR Purpura, Thrombocytopenic, Idiopathic/
281.	((autoimmun* OR idiopathic OR immun* OR purpura*) adj2 thrombocytopen*).mp. [mp=title, abstract, original title, name of substance word, subject heading word, floating sub-heading word, keyword heading word, organism supplementary concept word, protocol supplementary concept word, rare disease supplementary concept word, unique identifier, synonyms]
282.	(werlhof* adj1 disease).mp. [mp=title, abstract, original title, name of substance word, subject heading word, floating sub-heading word, keyword heading word, organism supplementary concept word, protocol supplementary concept word, rare disease supplementary concept word, unique identifier, synonyms]
283.	Raynaud disease.mp. OR Raynaud Disease/
284.	(raynaud adj1 (disease OR syndrom* OR phenomen*)).mp. [mp=title, abstract, original title, name of substance word, subject heading word, floating sub-heading word, keyword heading word, organism supplementary concept word, protocol supplementary concept word, rare disease supplementary concept word, unique identifier, synonyms]
285.	Retinal Vasculitis.mp. OR Retinal Vasculitis/
286.	(vasculitis adj1 retinal).mp. [mp=title, abstract, original title, name of substance word, subject heading word, floating sub-heading word, keyword heading word, organism supplementary concept word, protocol supplementary concept word, rare disease supplementary concept word, unique identifier, synonyms]
287.	Sarcoidosis/ OR sarcoid*.mp.
288.	((besnier-boeck-Schaumann OR besnier-boeck OR besnier* OR boeck* OR schaumann*) adj1 (syndrome or disease)).mp. [mp=title, abstract, original title, name of substance word, subject heading word, floating sub-heading word, keyword heading word, organism supplementary concept word, protocol supplementary concept word, rare disease supplementary concept word, unique identifier, synonyms]
289.	Shwartzman Phenomenon.mp. OR Shwartzman Phenomenon/
290.	((schwartzman OR shwartzman) adj1 (phenomenon OR reaction*)).mp. [mp=title, abstract, original title, name of substance word, subject heading word, floating sub-heading word, keyword heading word, organism supplementary concept word, protocol supplementary concept word, rare disease supplementary concept word, unique identifier, synonyms]
291.	Scleroderma, Diffuse.mp. OR Scleroderma, Diffuse/
292.	((scleroderma* OR sclerosis) adj2 (progressive OR sudden OR diffuse OR systemic)).mp. [mp=title, abstract, original title, name of substance word, subject heading word, floating sub-heading word, keyword heading word, organism supplementary concept word, protocol supplementary concept word, rare disease supplementary concept word, unique identifier, synonyms]
293.	Scleroderma, Localized.mp. OR Scleroderma, Localized/
294.	((limited OR local*) adj2 scleroderma*).mp. [mp=title, abstract, original title, name of substance word, subject heading word, floating sub-heading word, keyword heading word, organism supplementary concept word, protocol supplementary concept word, rare disease supplementary concept word, unique identifier, synonyms]
295.	(serum adj1 sickness*).mp. [mp=title, abstract, original title, name of substance word, subject heading word, floating sub-heading word, keyword heading word, organism supplementary concept word, protocol supplementary concept word, rare disease supplementary concept word, unique identifier, synonyms]
296.	Serum Sickness.mp. OR Serum Sickness/
297.	Sjogren's syndrome.mp. OR Sjogren's Syndrome/
298.	((sjogren* OR sicca) adj1 (disease OR syndrome)).mp. [mp=title, abstract, original title, name of substance word, subject heading word, floating sub-heading word, keyword heading word, organism supplementary concept word, protocol supplementary concept word, rare disease supplementary concept word, unique identifier, synonyms]
299.	Spondylitis, Ankylosing.mp. OR Spondylitis, Ankylosing/
300.	((spondyl* OR rheumatoid) adj1 ankylo*).mp. [mp=title, abstract, original title, name of substance word, subject heading word, floating sub-heading word, keyword heading word, organism supplementary concept word, protocol supplementary concept word, rare disease supplementary concept word, unique identifier, synonyms]
301.	(bechterew* adj1 disease).mp. [mp=title, abstract, original title, name of substance word, subject heading word, floating sub-heading word, keyword heading word, organism supplementary concept word, protocol supplementary concept word, rare disease supplementary concept word, unique identifier, synonyms]
302.	Stiff-Person Syndrome.mp. OR Stiff-Person Syndrome/
303.	((stiff* OR startle OR moersch woltmann OR moersch*) adj2 syndrome*).mp. [mp=title, abstract, original title, name of substance word, subject heading word, floating sub-heading word, keyword heading word, organism supplementary concept word, protocol supplementary concept word, rare disease supplementary concept word, unique identifier, synonyms]
304.	(still* adj1 disease).mp. [mp=title, abstract, original title, name of substance word, subject heading word, floating sub-heading word, keyword heading word, organism supplementary concept word, protocol supplementary concept word, rare disease supplementary concept word, unique identifier, synonyms]
305.	Still Disease, Adult-Onset.mp. OR Still's Disease, Adult-Onset/
306.	(systemic adj1 vasculiti*).mp. [mp=title, abstract, original title, name of substance word, subject heading word, floating sub-heading word, keyword heading word, organism supplementary concept word, protocol supplementary concept word, rare disease supplementary concept word, unique identifier, synonyms]
307.	Systemic Vasculitis.mp. OR Systemic Vasculitis/
308.	Takayasu Arteritis.mp. OR Takayasu Arteritis/
309.	((arteriti* OR aortitis OR disease) adj1 (takayasu* OR syndome OR female)).mp. [mp=title, abstract, original title, name of substance word, subject heading word, floating sub-heading word, keyword heading word, organism supplementary concept word, protocol supplementary concept word, rare disease supplementary concept word, unique identifier, synonyms]
310.	Thromboangiitis Obliterans.mp. OR Thromboangiitis Obliterans/
311.	(thromboangi* adj1 obliterans).mp. [mp=title, abstract, original title, name of substance word, subject heading word, floating sub-heading word, keyword heading word, organism supplementary concept word, protocol supplementary concept word, rare disease supplementary concept word, unique identifier, synonyms]
312.	(buerger* adj1 disease).mp. [mp=title, abstract, original title, name of substance word, subject heading word, floating sub-heading word, keyword heading word, organism supplementary concept word, protocol supplementary concept word, rare disease supplementary concept word, unique identifier, synonyms]
313.	Thyroiditis, Autoimmune.mp. OR Thyroiditis, Autoimmune/
314.	((autoimmune OR lympho*) adj1 thyroiditi*).mp. [mp=title, abstract, original title, name of substance word, subject heading word, floating sub-heading word, keyword heading word, organism supplementary concept word, protocol supplementary concept word, rare disease supplementary concept word, unique identifier, synonyms]
315.	(autoimmune adj1 (hypothyroiditi* OR hyperthyroiditi*)).mp. [mp=title, abstract, original title, name of substance word, subject heading word, floating sub-heading word, keyword heading word, organism supplementary concept word, protocol supplementary concept word, rare disease supplementary concept word, unique identifier, synonyms]
316.	Undifferentiated Connective Tissue Diseases.mp. OR Undifferentiated Connective Tissue Diseases/
317.	(undifferentiated connective tissue adj1 disease*).mp. [mp=title, abstract, original title, name of substance word, subject heading word, floating sub-heading word, keyword heading word, organism supplementary concept word, protocol supplementary concept word, rare disease supplementary concept word, unique identifier, synonyms]
318.	Uveomeningoencephalitic Syndrome.mp. OR Uveomeningoencephalitic Syndrome/
319.	((syndrome OR disease) adj1 (uveomeningoencephalitic OR VKH OR vogt* OR vogt koyanagi harada)).mp. [mp=title, abstract, original title, name of substance word, subject heading word, floating sub-heading word, keyword heading word, organism supplementary concept word, protocol supplementary concept word, rare disease supplementary concept word, unique identifier, synonyms]
320.	uveomeningoencephaliti*.mp.
321.	(vasculiti* OR angiiti*).mp. [mp=title, abstract, original title, name of substance word, subject heading word, floating sub-heading word, keyword heading word, organism supplementary concept word, protocol supplementary concept word, rare disease supplementary concept word, unique identifier, synonyms]
322.	Vasculitis.mp. OR Vasculitis/
323.	Vasculitis, Central Nervous System.mp. OR Vasculitis, Central Nervous System/
324.	((vasculiti* OR angitiis OR arteritis) adj2 (central nervous system OR CNS OR cerebral OR granulomatous)).mp. [mp=title, abstract, original title, name of substance word, subject heading word, floating sub-heading word, keyword heading word, organism supplementary concept word, protocol supplementary concept word, rare disease supplementary concept word, unique identifier, synonyms]
325.	Vasculitis, Leukocytoclastic, Cutaneous.mp. OR Vasculitis, Leukocytoclastic, Cutaneous/
326.	((vasculiti* OR angiiti*) adj2 (cutan* OR skin OR leukocytoclastic OR hypersensitivity)).mp. [mp=title, abstract, original title, name of substance word, subject heading word, floating sub-heading word, keyword heading word, organism supplementary concept word, protocol supplementary concept word, rare disease supplementary concept word, unique identifier, synonyms]
327.	(leukoderma OR leucoderma OR vitiligo).mp. [mp=title, abstract, original title, name of substance word, subject heading word, floating sub-heading word, keyword heading word, organism supplementary concept word, protocol supplementary concept word, rare disease supplementary concept word, unique identifier, synonyms]
328.	vitiligo.mp. OR Vitiligo/
329.	90 OR 91 OR 92 OR 93 OR 94 OR 95 OR 96 OR 97 OR 98 OR 99 OR 100 OR 101 OR 102 OR 103 OR 104 OR 105 OR 106 OR 107 OR 108 OR 109 OR 110 OR 111 OR 112 OR 113 OR 114 OR 115 OR 116 OR 117 OR 118 OR 119 OR 120 OR 121 OR 122 OR 123 OR 124 OR 125 OR 126 OR 127 OR 128 OR 129 OR 130 OR 131 OR 132 OR 133 OR 134 OR 135 OR 136 OR 137 OR 138 OR 139 OR 140 OR 141 OR 142 OR 143 OR 144 OR 145 OR 146 OR 147 OR 148 OR 149 OR 150 OR 151 OR 152 OR 153 OR 154 OR 155 OR 156 OR 157 OR 158 OR 159 OR 160 OR 161 OR 162 OR 163 OR 164 OR 165 OR 166 OR 167 OR 168 OR 169 OR 170 OR 171 OR 172 OR 173 OR 174 OR 175 OR 176 OR 177 OR 178 OR 179 OR 180 OR 181 OR 182 OR 183 OR 184 OR 185 OR 186 OR 187 OR 188 OR 189 OR 190 OR 191 OR 192 OR 193 OR 194 OR 195 OR 196 OR 197 OR 198 OR 199 OR 200 OR 201 OR 202 OR 203 OR 204 OR 205 OR 206 OR 207 OR 208 OR 209 OR 210 OR 211 OR 212 OR 213 OR 214 OR 215 OR 216 OR 217 OR 218 OR 219 OR 220 OR 221 OR 222 OR 223 OR 224 OR 225 OR 226 OR 227 OR 228 OR 229 OR 230 OR 231 OR 232 OR 233 OR 234 OR 235 OR 236 OR 237 OR 238 OR 239 OR 240 OR 241 OR 242 OR 243 OR 244 OR 245 OR 246 OR 247 OR 248 OR 249 OR 250 OR 251 OR 252 OR 253 OR 254 OR 255 OR 256 OR 257 OR 258 OR 259 OR 260 OR 261 OR 262 OR 263 OR 264 OR 265 OR 266 OR 267 OR 268 OR 269 OR 270 OR 271 OR 272 OR 273 OR 274 OR 275 OR 276 OR 277 OR 278 OR 279 OR 280 OR 281 OR 282 OR 283 OR 284 OR 285 OR 286 OR 287 OR 288 OR 289 OR 290 OR 291 OR 292 OR 293 OR 294 OR 295 OR 296 OR 297 OR 298 OR 299 OR 300 OR 301 OR 302 OR 303 OR 304 OR 305 OR 306 OR 307 OR 308 OR 309 OR 310 OR 311 OR 312 OR 313 OR 314 OR 315 OR 316 OR 317 OR 318 OR 319 OR 320 OR 321 OR 322 OR 323 OR 324 OR 325 OR 326 OR 327 OR 328
330.	Epidemiologic Studies.mp. OR Epidemiologic Studies/
331.	(epidemiolog* adj1 stud*).mp. [mp=title, abstract, original title, name of substance word, subject heading word, floating sub-heading word, keyword heading word, organism supplementary concept word, protocol supplementary concept word, rare disease supplementary concept word, unique identifier, synonyms]
332.	Case-Control Studies.mp. OR Case-Control Studies/
333.	(case* adj2 stud*).mp. [mp=title, abstract, original title, name of substance word, subject heading word, floating sub-heading word, keyword heading word, organism supplementary concept word, protocol supplementary concept word, rare disease supplementary concept word, unique identifier, synonyms]
334.	Cohort Studies.mp. OR Cohort Studies/
335.	cohort*.mp.
336.	Follow-Up Studies.mp. OR Follow-Up Studies/
337.	(follow* adj2 stud*).mp. [mp=title, abstract, original title, name of substance word, subject heading word, floating sub-heading word, keyword heading word, organism supplementary concept word, protocol supplementary concept word, rare disease supplementary concept word, unique identifier, synonyms]
338.	Longitudinal Studies/ OR Longitudinal Studies.mp.
339.	(longitudin* adj1 stud*).mp. [mp=title, abstract, original title, name of substance word, subject heading word, floating sub-heading word, keyword heading word, organism supplementary concept word, protocol supplementary concept word, rare disease supplementary concept word, unique identifier, synonyms]
340.	Prospective Studies.mp. OR Prospective Studies/
341.	(prospectiv* adj1 stud*).mp. [mp=title, abstract, original title, name of substance word, subject heading word, floating sub-heading word, keyword heading word, organism supplementary concept word, protocol supplementary concept word, rare disease supplementary concept word, unique identifier, synonyms]
342.	prospectiv*.mp.
343.	Retrospective Studies.mp. OR Retrospective Studies/
344.	(retrospective adj1 stud*).mp. [mp=title, abstract, original title, name of substance word, subject heading word, floating sub-heading word, keyword heading word, organism supplementary concept word, protocol supplementary concept word, rare disease supplementary concept word, unique identifier, synonyms]
345.	retrospectiv*.mp.
346.	Comparative Study.mp. OR Comparative Study/
347.	(comparati* adj1 stud*).mp. [mp=title, abstract, original title, name of substance word, subject heading word, floating sub-heading word, keyword heading word, organism supplementary concept word, protocol supplementary concept word, rare disease supplementary concept word, unique identifier, synonyms]
348.	Case Reports.mp. OR Case Reports/
349.	(case adj1 (stud* OR report* OR histor*)).mp. [mp=title, abstract, original title, name of substance word, subject heading word, floating sub-heading word, keyword heading word, organism supplementary concept word, protocol supplementary concept word, rare disease supplementary concept word, unique identifier, synonyms]
350.	(case adj2 serie*).mp. [mp=title, abstract, original title, name of substance word, subject heading word, floating sub-heading word, keyword heading word, organism supplementary concept word, protocol supplementary concept word, rare disease supplementary concept word, unique identifier, synonyms]
351.	(observation* adj1 (stud* OR cohort)).mp. [mp=title, abstract, original title, name of substance word, subject heading word, floating sub-heading word, keyword heading word, organism supplementary concept word, protocol supplementary concept word, rare disease supplementary concept word, unique identifier, synonyms]
352.	Observation/ OR observation*.mp.
353.	Multivariate Analysis.mp. OR Multivariate Analysis/
354.	(multivaria* adj1 analys*).mp. [mp=title, abstract, original title, name of substance word, subject heading word, floating sub-heading word, keyword heading word, organism supplementary concept word, protocol supplementary concept word, rare disease supplementary concept word, unique identifier, synonyms]
355.	Cross-Sectional Studies.mp. OR Cross-Sectional Studies/
356.	((cross* OR prevalence) adj2 (stud* OR analys* OR survey*)).mp. [mp=title, abstract, original title, name of substance word, subject heading word, floating sub-heading word, keyword heading word, organism supplementary concept word, protocol supplementary concept word, rare disease supplementary concept word, unique identifier, synonyms]
357.	(disease frequency adj2 survey*).mp. [mp=title, abstract, original title, name of substance word, subject heading word, floating sub-heading word, keyword heading word, organism supplementary concept word, protocol supplementary concept word, rare disease supplementary concept word, unique identifier, synonyms]
358.	Risk Factors.mp. OR Risk Factors/
359.	(risk adj2 (factor* OR population)).mp. [mp=title, abstract, original title, name of substance word, subject heading word, floating sub-heading word, keyword heading word, organism supplementary concept word, protocol supplementary concept word, rare disease supplementary concept word, unique identifier, synonyms]
360.	groups.mp.
361.	(register-based OR (register* adj based)).mp. [mp=title, abstract, original title, name of substance word, subject heading word, floating sub-heading word, keyword heading word, organism supplementary concept word, protocol supplementary concept word, rare disease supplementary concept word, unique identifier, synonyms]
362.	Registries/ OR Registries.mp.
363.	regist*.mp.
364.	(population based OR population-based).mp. [mp=title, abstract, original title, name of substance word, subject heading word, floating sub-heading word, keyword heading word, organism supplementary concept word, protocol supplementary concept word, rare disease supplementary concept word, unique identifier, synonyms]
365.	330 OR 331 OR 332 OR 333 OR 334 OR 335 OR 336 OR 337 OR 338 OR 339 OR 340 OR 341 OR 342 OR 343 OR 344 OR 345 OR 346 OR 347 OR 348 OR 349 OR 350 OR 351 OR 352 OR 353 OR 354 OR 355 OR 356 OR 357 OR 358 OR 359 OR 360 OR 361 OR 362 OR 363 OR 364
366.	89 AND 329 AND 365
