# Supplementary material for: Psychiatric medications and the risk of autoimmune and immune-mediated inflammatory diseases: A systematic review and meta-analysis of observational studies
Source: PLoS One. 2023 Feb 28;18(2):e0281979. doi: 10.1371/journal.pone.0281979 (PMC9974122; doi:10.1371/journal.pone.0281979)
Supplement: S2 File — (RTF) [file pone.0281979.s002.rtf]

S2 
Search strategy SCOPUS
Search from the beginning of indexing until 28.11.2021: 3408 documents
TITLE-ABS-KEY(carbamazepine OR oxcarbazepine OR "Valproic acid" OR valproate OR lamotrigine OR "Antipsychotic Agents" OR antipsychotic* OR lithium OR antidepressive OR antidepressant* OR desipramine OR imipramine OR clomipramine OR opipramol OR trimipramine OR lofepramine OR dibenzazepine* OR dibenzepin OR amitriptyline OR nortriptyline OR Protriptyline OR Doxepin OR Iprindole OR melitracen OR butriptyline OR dosuleptin OR dothiepin OR amoxapine OR dimetacrine OR amineptine OR maprotiline OR quinupramine OR "tricyclic antidepressant*" OR "serotonin uptake inhibitor*" OR SSRI OR zimelidine OR fluoxetine OR citalopram OR paroxetine OR sertraline OR alaproclate OR fluvoxamine OR etoperidone OR escitalopram OR "monoamine oxidase inhibitor*" OR isocarboxazid OR nialamide OR phenelzine OR tranylcypromine OR iproclozide OR moclobemide OR toloxatone OR oxitriptan OR mianserin OR nomifensine OR trazodone OR nefazodone OR minaprine OR bifemelane OR viloxazine OR oxaflozane OR mirtazapine OR bupropion OR medifoxamine OR tianeptine OR pivagabine OR venlafaxine OR milnacipran OR reboxetine OR gepirone OR duloxetine OR agomelatine OR desvenlafaxine OR vilazodone OR vortioxetine OR chlorpromazine OR haloperidol OR perphenazine OR fluphenazine OR risperidone OR olanzapine OR quetiapine OR ziprasidone OR aripiprazole OR paliperidone OR lurasidone OR asenapine OR clozapine OR iloperidone OR levomepromazine OR perazine OR zuclopenthixol OR clopenthixol) AND TITLE-ABS-KEY( (addison* W/1 disease*) OR (adren* W/1 "insufficienc* OR autoimmun*") OR ("alopecia W/1 areata") OR ("alopecia W/1 totalis" OR "alopecia W/1 universalis") OR ("anemia* OR anaemia* OR hemoly* OR haemoly*" W/2 autoimmun*) OR "(addison* OR pernicious) W/1 (anaemia OR anemia)" OR "autoimmun* W/1 gastriti*" OR "(goodpasture* OR anti-GBM OR GBM OR anti-glomerular) W/1 (disease OR syndrome)" OR "(ANCA OR anti-neutrophil OR cytoplasmic OR pausi-immune) W/1 (antibody OR associated OR vasculiti*)" OR "NMDA* OR Anti-N-Methyl-D-Aspartate W/2 encephaliti*" OR "(hughes OR antiphospholipid OR phospholipid OR anti-phospholipid) W/1 syndrome*" OR aortiti* OR arteriti* OR "arterial W/1 inflammation" OR "(arthriti* OR fever* OR polyarthriti* OR polymyalgia OR inflammator*) W/1 (psoria* OR reactive OR rheumat* OR juvenil*)" OR "arthus W/1 (phenomenon OR reaction)" OR autoantibod* OR autoimmun* W/1 (disease* OR disorder*) OR "(autoimmun* OR immun*) W/2 (neurologic OR nervous)" OR "(autoimmun* OR lympho*) W/1 (hypophysiti* OR panhypophysiti* OR adenohypophysiti*)" OR "autoimmun* W/2 lymphoproliferativ*" OR Canale-Smith OR "pancreatiti*" OR autoimmun* OR autoantibod* OR autoinflammat* OR "autoimmun* W/2 (angioedema* OR edema* OR skin OR cutis OR cutaneous OR urticaria)" OR "autoimmun* W/2 (neutropenia OR lymphocytopenia OR lymphopenia)" OR "autoimmun* W/2 (myocardit* OR heart)" OR "autoimmun* W/2 (oophoritis OR orchitis OR gonad*)" OR "autoimmun* W/2 (polyglandular OR polyendocrinopath*)" OR "autoimmun* W/2 (retinopathy OR uveitis OR eye)" OR "behcet* W/1 (syndrome OR disease*)" OR "triple symptom W/2 compl*" OR "(celiac OR coeliac OR gluten*) W/1 (enteropath* OR disease OR sprue)" OR "sclerosing ADJ1 cholangiti*" OR "eosinophilic W/2 granulomato* W/2 polyangiiti*" OR "churg W/1 strauss" OR "syndrome W/1 cogan*" OR "(collagenous OR lymphocytic OR microscopic) W/1 colitis" OR "(ulcerative OR gravis) W/1 colitis" OR "inflammat* W/1 bowel" OR "granulomatous W/1 (colitis OR enteritis)" OR "idiopathic proctocolitis" OR IBD OR UC OR "connective W/1 tissue W/1 (disease* OR disorder*)" OR "(CRST OR CREST) W/1 syndrom*" OR "(crohn* OR granulomatous) W/1 (disease* OR enterocolitis OR colitis OR enteritis)" OR ileocolitis OR "inflammat* W/2 bowel" OR "(terminal OR regional) W/1 ileiti*" OR "demyelinating W/3 autoimmune W/3 (CNS OR brain OR cerebral OR spinal OR central nervous)" OR "dermatitis W/1 herpetiformis" OR "duhring* W/1 disease" OR dermatomyositis OR dermatopolymyositis OR "(myositis OR dermatomyositis) W/1 (juvenile OR polymyositis* OR childhood)" OR "(autoimmun* OR type 1) W/2 diabetes" OR "insulin-dependent diabetes" OR "(disseminated OR autoimmun*) W/2 encephalomyeliti*" OR endarteriti* OR "felt* W/1 syndrome" OR "(arteriti* OR aortiti*) W/1 (giant cell OR temporal OR cranial)" OR "horton* W/1 disease" OR IGA OR "glomerulonephriti* OR kindney W/2 autoimmun*" OR "(immunog* OR membranous) W/1 (nephropathy OR glomerulonephriti*)" OR "heymann W/1 nephritis" OR "granulomatosis W/1 polyangiiti*" OR "wegener* W/1 (granuloma* OR polyang*)" OR "(basedow* OR grave*) W/1 disease" OR "guillain W/1 barre" OR "inflammatory W/1 (polyneuropath* OR polyradiculoneuropath*)" OR "hashimoto* W/1 (disease OR struma OR thyroiditi*)" OR "autoimmun* W/1 hepatiti*" OR "hypersomn* W/1 idiopathic" OR "(IgG4-related OR immunoglobulin G4* OR IgG4*) W/2 (disease OR syndrome)" OR "lambert* W/1 (eaton OR myasthenic)" OR LADA OR "latent W/2 diabetes" OR "lichen W/2 planus" OR "lichen* W/1 eruption*" OR "dermatos* W/2 linear W/2 IgA" OR "biliary W/1 (cirrhos*)" OR PBC OR "lupus W/1 (cutaneous OR discoid OR erythematosus OR nephritis OR skin OR vasculitis)" OR "libman W0 sacks" OR "lupus W/1 (glomerulonephriti* OR nephriti*)" OR "(central nervous system OR CNS OR meningoencephaliti*) W/2 lupus" OR "(atrophic OR degos* OR kohlmeier*) W/1 (papuloses OR disease)" OR mastocytos* OR "mast cell W/2 (syndrome OR disease*)" OR "polyangiiti* W/1 microscopic" OR "(kawasaki OR mucocutaneous lymph node) W/1 (disease* OR syndrome*)" OR "(multiple OR disseminated) W/1 (sclerosis OR myelitis)" OR "(myastheni*) W/1 (gravis OR disease OR syndrome)" OR myositi* OR narcolepsy OR "(narcoleptic OR narcolepsy-cataplexy OR gelineau*) W/1 syndrom*" OR "paroxysmal W/1 sleep" OR "(opsoclonus* OR encephalopath*) W/1 myoclon*" OR "dancing W/1 (eyes OR feet)" OR pemphigoid* OR (bullous W/1 pemphigoid) OR "pemphigus W/1 (foliaceus OR vulgaris)" OR phlebiti* OR periphlebiti* OR POEMS OR takatsuki* OR Crow-Fukase OR "polyneuropath* W/1 organomegal*" OR "(periarteritis OR polyarteriti* OR arteriti*) W/1 (nodosa OR essential OR necrotizing)" OR "autoimmun* W/3 (polyendocrinopath* OR polyglandular OR oolyendocrine)" OR "AIRE W/1 deficienc*" OR "schmidt* W/1 syndrome" OR "polymyalgia W/1 rheumatica" OR "forestier* W/1 certonciny" OR "rheumatism W/1 peri*" OR "(pseudopolyarthriti* OR pseudopolyarthriti*) W/1 rhizomelic" OR polymyositi* OR "(multiple OR idiopathic OR ossificans) W/1 (myositi* OR polymyositi*)" OR CIDP OR "(polyneuropath* OR polyradiculopath* OR polyradiculoneuropath*) W/2 inflammat*" OR "psoria* OR (pustul* W/1 palm*)" OR "(henoch OR schoenlein OR anaphylactoid OR allergic OR rheumatoid OR hemorrhagica OR nonthrombo*) W/2 purpura*" OR "(autoimmun* OR idiopathic OR immun* OR purpura*) W/2 thrombocytopen*" OR "werlhof* W/1 disease" OR "raynaud W/1 (disease OR syndrom* OR phenomen*)" OR "vasculitis W/1 retinal OR sarcoid*" OR "(besnier* OR boeck* OR schaumann*) W/1 (syndrome OR disease)" OR "(schwartzman OR shwartzman) W/1 (phenomenon OR reaction*)" OR "(scleroderma* OR sclerosis) W/2 (progressive OR sudden OR diffuse OR systemic)" OR "(limited OR local*) W/2 scleroderma*" OR "serum W/1 sickness*" OR "(sjogren* OR sicca) W/1 (disease OR syndrome)" OR "(spondyl* OR rheumatoid) W/1 ankylo*" OR "bechterew* W/1 disease" OR "(stiff* OR startle OR moersch woltmann OR moersch*) W/2 syndrome*" OR "still* W/1 disease" OR "systemic W/1 vasculiti*" OR "(arteriti* OR aortitis OR disease) W/1 (takayasu* OR syndome OR female)" OR "thromboangi* W/1 obliterans" OR "buerger* W/1 disease" OR "(autoimmune OR lympho*) W/1 thyroiditi*" OR "autoimmune W/1 (hypothyroiditi* OR hyperthyroiditi*)" OR "undifferentiated connective tissue W/1 disease*" OR "(syndrome OR disease) W/1 (uveomeningoencephalitic OR VKH OR vogt* OR vogt koyanagi harada)" OR uveomeningoencephaliti* OR vasculiti* OR angiiti* OR "(vasculiti* OR angitiis OR arteritis) W/2 (central nervous system OR CNS OR cerebral OR granulomatous)" OR "(vasculiti* OR angiiti*) W/2 (cutan* OR skin OR leukocytoclastic OR hypersensitivity)" OR leukoderma OR leucoderma OR vitiligo) AND (ALL ( "epidemiolog* W/1 stud*" OR "case* W/2 stud*" OR cohort* OR "follow* W/2 stud*" OR "longitudin* W/1 stud*" OR "prospectiv* W/1 stud*" OR prospectiv* OR "retrospective W/1 stud*" OR retrospectiv* OR "comparati* W/1 stud*" OR "case W/1 (stud* OR report* OR histor*)" OR "case W/2 serie*" OR "observation* W/1 (stud* OR cohort)" OR observation* OR "multivaria* W/1 analys*" OR "(cross* OR prevalence) W/2 (stud* OR analys* OR survey*)" OR "disease frequency W/2 survey*" OR "risk W/2 (factor* OR population)" OR groups OR "register* W/0 based" OR regist* OR "population based") )
