# Supplementary material for: Psychiatric medications and the risk of autoimmune and immune-mediated inflammatory diseases: A systematic review and meta-analysis of observational studies
Source: PLoS One. 2023 Feb 28;18(2):e0281979. doi: 10.1371/journal.pone.0281979 (PMC9974122; doi:10.1371/journal.pone.0281979)
Supplement: S4 File — (RTF) [file pone.0281979.s004.rtf]

S4
Newcastle-Ottawa Scale assessment of case-control and cohort studies for risk of bias
Table 1A. Newcastle-Ottawa Scale assessment of case-control studies for risk of bias
Author, year	Selection	Comparability	Exposure		
	Case definition	Representativeness of the cases	Selection of controls	Definition of controls	Comparability of cases and controls on the basis of study design or analysis	Ascertainment of exposure	Same method of ascertainment and controls
	Non-response rate	Total score	
Bastuji-Garin 1996	★	★	-	★	★★	-	★	-	6	
Bastuji-Garin 2011	★	★	-	★	★★	-	★	-	6	
Boden 2012	★	★	★	★	★★	★	★	★	9	
Bonderup 2014	★	★	★	★	★★	★	★	★	9	
Brauchli 2009	★	★	★	★	★★	★	★	★	9	
Fernández-Bañares 2013	★	★	-	★	★	-	★	-	5	
Fernández-Bañares 2007	★	★	-	-	★	-	★	★	5	
Garbe 2012	★	★	-	★	★	-	★	-	5	
Gasse 2008	★	★	★	★	★★	★	★	★	9	
Grönhagen 2012	★	★	-	★	★	-	★	-	5	
Lin 2017	★	★	★	-	★	★	★	★	7	
Ljung 2012	★	★	★	-	★	★	★	★	7	
Lloyd-Lavery 2013	★	★	★	★	★★	★	★	★	9	
Masclee 2015	★	★	★	★	★★	★	★	★	9	
Miller 2001	★	-	-	★	★★	-	★	-	5	
Norgaard 2006	★	★	★	★	★★	★	★	★	9	
Norgaard 2007	★	★	-	★	-	-	★	★	5	
Pascua 2010	★	★	★	★	★★	★	★	★	9	
Rosenberg 2017	★	-	-	★	★★	-	★	★	6	
Schoonen 2010	★	-	-	★	-	★	★	★	5	
Vallerand 2019	★	★	★	★	★★	★	★	★	9	
Varpuluoma 2019	★	★	-	★	★★	★	★	★	8	
Verhaegh 2016	★	★	★	★	★★	★	★	★	9	
Weimers 2021	★	★	★	★	-	★	★	★	7	


Table 1B. Newcastle-Ottawa Scale assessment of cohort studies for risk of bias


Author, year	Selection	Comparability	Outcome		
	Population-based representativeness


	Selection of the nonexposed  cohort	Ascertainment of exposure
	Outcome not present at start	Comparability of cohorts on the basis of the design or analysis	Assessment of outcome
	Was follow-up long enough for outcomes to occur
	Adequacy of follow up of cohorts	Total score


Total score


	
Mirza 2021	-	★	-	★	★★	-	★	-	5	
Nielsen 2015	★	★	★	★	★	★	★	★	8	
Sparks 2021	-	★	-	★	★★	-	★	★	6	
Roberts 2018	-	★	-	★	★★	-	★	-	5	
Tzeng 2021	★	★	★	★	★★	★	★	★	9	
Vallerand 2018	★	★	★	★	★★	★	★	★	9	
Vallerand 2019	★	★	★	★	★★	★	★	★	9	
