# Supplementary material for: Psychiatric medications and the risk of autoimmune and immune-mediated inflammatory diseases: A systematic review and meta-analysis of observational studies
Source: PLoS One. 2023 Feb 28;18(2):e0281979. doi: 10.1371/journal.pone.0281979 (PMC9974122; doi:10.1371/journal.pone.0281979)
Supplement: S5 File — (RTF) [file pone.0281979.s005.rtf]

S5
Data used in meta-analyses

The odds of microscopic colitis after exposure to any SSRI.	
Study	OR	95 % CI LB	95 % CI UB	Drug use / exposure definition	
1Fernandez-Banares et al. 2007	CC 21
LC 37.7
Both 28.31	CC 2.5
LC 4.7
Both 6.38	CC 177
LC 304
Both 125.61	Chronic use of > 3 days/week for a period longer than 2 weeks before the questionnaire.	
Pascua et al. 2010	0.55	0.20	1.55	Use within 12 months from diagnosis.	
1Bonderup 2014	CC 1.59
LC 2.89
Both 2.14	CC 1.45
LC 2.61
Both 1.19	CC 1.74
LC 3.20
Both 3.85	Prescription of the drug within 1 year preceding the diagnosis.	
Masclee et al. 2015	5.4	2.8	10.4	Current use within 1 year before the index date.	
Verhaegh et al. 2016	2.03	1.58	2.61	Current use.	
Weimers et al. 2021	1.47	1.44	1.50	Current use. Within one year before the index date.	
OR = Odds ratio; CI = Confidence interval; LB = Lower bound; UB = Upper bound; CC = Collagenous colitis; LC = Lymphocytic colitis.
1The studies by Fernandez-Banares and Bonderup didn't report OR for MC, but for their subtypes CC and LC. For these studies to be included, both estimates were combined.	

The odds of collagenous colitis after exposure to any SSRI.	
Study	OR	95 % CI LB	95 % CI UB	Drug use / exposure definition	
Fernandez-Banares et al. 2007	21	2.5	177	Chronic use of > 3 days/week for a period longer than 2 weeks before the questionnaire.	
Bonderup et al. 2014	1.59	1.45	1.74	Prescription of the drug within 1 year preceding the diagnosis.	
Verhaegh et al. 2016	1.00	0.61	1.65	Current use.	
OR = Odds ratio; CI = Confidence interval; LB = Lower bound; UB = Upper bound.	

The odds of lymphocytic colitis after exposure to any SSRI.	
Study	OR	95 % CI LB	95 % CI UB	Drug use / exposure definition	
Fernandez-Banares et al. 2007	37.7	4.7	304	Chronic use of > 3 days/week for a period longer than 2 weeks before the questionnaire.	
Bonderup et al. 2014	2.89	2.61	3.20	Prescription of the drug within 1 year preceding the diagnosis.	
Verhaegh et al. 2016	2.28	1.43	3.63	Current use.	
OR = Odds ratio; CI = Confidence interval; LB = Lower bound; UB = Upper bound.	

The odds of psoriasis after exposure to any SSRI.	
Study	OR	95 % CI LB	95 % CI UB	Drug use / exposure definition	
Brauchli et al. 2009	0.93	0.80	1.09	Current use and in long-term.	
Tzeng et al. 2021	0.67	0.54	0.84	Any antidepressant use.	
OR = Odds ratio; CI = Confidence interval; LB = Lower bound; UB = Upper bound.	

The odds of bullous pemphigoid after exposure to antipsychotics.	
Study	OR	95 % CI LB	95 % CI UB	Drug use / exposure definition	
Bastuji-Garin et al.  1996	1.9	0.95	3.8	Over three months, any dose.	
Bastuji-Garin et al.  2011	1.60	0.89	2.75	Over three months, any dose.	
Lloyd-Lavery et al. 2013	3.3	0.7	20.7	Any use before diagnosis.	
OR = Odds ratio; CI = Confidence interval; LB = Lower bound; UB = Upper bound.	

Odds of pancreatitis after exposure to any SSRI.	
Study	OR	95 % CI LB	95 % CI UB	Drug use / exposure definition	
Ljung 2012	1.5	1.4	1.7	Current use, within 1-114 days.	
Norgaard 2007	1.2	1.0	1.5	Current use.	
OR = Odds ratio; CI = Confidence interval; LB = Lower bound; UB = Upper bound.	
